# Ciliogenesis-associated Kinase 1 Promotes Breast Cancer Cell Proliferation and Chemoresistance via Phosphorylating ERK1

**DOI:** 10.7150/ijbs.87442

**Published:** 2024-04-08

**Authors:** Yanling He, Xinyuan Zhang, Weijun Pan, Jiebiao Zhang, Weiliang Zhu, Jian Zhang, Jian Shi

**Affiliations:** 1 Department of Pathology, School of Basic Medical Science, Southern Medical University, Guangzhou 510515, Guangdong, China.; 2 Department of Oncology, Zhujiang Hospital, Southern Medical University, Guangzhou, Guangdong, China.

**Keywords:** Breast cancer, CILK1, ERK1, Proliferation, Chemoresistance

## Abstract

Ciliogenesis-associated kinase 1 (CILK1) plays a key role in the ciliogenesis and ciliopathies. It remains totally unclear whether CILK1 is involved in tumor progression and therapy resistance. Here, we report that the aberrant high-expression of CILK1 in breast cancer is required for tumor cell proliferation and chemoresistance. Two compounds, CILK1-C30 and CILK1-C28, were identified with selective inhibitory effects towards the Tyr-159/Thr-157 dual-phosphorylation of CILK1, pharmacological inhibition of CILK1 significantly suppressed tumor cell proliferation and overcame chemoresistance in multiple experimental models. Large-scale screen of CILK1 substrates confirmed that the kinase directly phosphorylates ERK1, which is responsible for CILK1-mediated oncogenic function. CILK1 is also indicated to be responsible for the chemoresistance of small-cell lung cancer cells. Our data highlight the importance of CILK1 in cancer, implicating that targeting CILK1/ERK1 might offer therapeutic benefit to cancer patients.

## Introduction

Ciliogenesis-associated kinase 1 (CILK1) is a serine/threonine protein kinase that originally identified from the gut 1. CILK1 protein shares significant sequential and structural homology with CDK1 at N-terminal catalytic domains 2. Mutations in the human *CILK1* gene have been associated with ciliopathies, a group of human genetic disorders with defects in the primary cilia. Primary cilia are able to influence quite a few biological processes including cell-cycle entry, proliferation, DNA damage/repair, autophagy and apoptosis by directly sensing and transducing signals in the microenvironment, they have been suggested with oncogenic or tumor suppressor function under diverse cellular context. Although CILK1 plays an essential role in regulating the function of cilia, its functional role in cancer is poorly defined 3-6. CILK1 protein consists of a serine/threonine kinase catalytic domain, a catalytic specificity loop, two phosphorylation activation loops, two nuclear-localization-signal (NLS) sites, and a proline-rich region 7. The c-terminal domain of CILK1 has been shown to be indispensable for the substrate recognition similar as that of MAP kinases 8. CILK1 is one of mammalian kinases with auto-phosphorylation activity, the dual phosphorylation of the TDY motif confers it with full catalytic activity 9. Here, we aim to explore the functional role of CILK1 in tumorgenicity and therapy resistance, identify its downstream signals, and develop the small-molecule compounds that are able to pharmacologically target the protein.

Chemotherapy remains the major treatment for several kinds of aggressive cancer that lack of target- or immune-therapies, including Triple-Negative Breast Cancer (TNBC) and Small-Cell Lung Cancer (SCLC). TNBC is a group of highly heterogeneous tumors, with high rate of recurrence. Due to the lack of specific drug targets, there is no effective targeted therapy for TNBC so far 10. Paclitaxel-based neoadjuvant chemotherapy is recommended as standard treatment for TNBC patients 11, 12. Despite pleasurable initial response, paclitaxel resistance remains a major therapeutic obstacle for TNBC patients 13, 14. Compared with non-small cell lung cancer (NSCLC), small cell lung cancer (SCLC) has a worse prognosis, earlier development of metastasis and recurrence. There is absence of clear biomarker-driven treatment options in SCLC, the patients are normally treated with a combination of platinum-based agent and etoposide 15, but the development of drug resistance is universal 16, drug resistance is also a major impediment to improvement of overall survival of patients with SCLC. A previous study suggested that the increased CILK1 expression in response to protein deprivation protects cell from apoptosis and maintains growth potential under nutritional stress 17, but it remains completely unclear whether it is involved in the chemotherapy resistance.

Here, our data uncover the key role of CILK1 in the tumor progression and chemoresistance. We employed liquid chromatography-coupled tandem mass spectrometry (LC-MS/MS)-based analysis to globally characterize downstream signals of CILK1 and reveal the CILK1/ERK1 signaling. Two small-molecule compounds are identified as the selective inhibitors of CILK1 and demonstrated to show robust anti-tumor efficacy in combination with chemotherapy.

## Materials and Methods

### Cell lines

Breast cancer cell lines MDA-MB-231, BT474 and MCF7 were cultured in DMEM medium supplemented with 10% fetal bovine serum (FBS) (Gibco, USA), 1% penicillin (100 mg/ml) and streptomycin (100 mg/ml) (Thermo Fisher Scientific, USA); BT549 and T47D cells were cultured in RPMI-1640 medium (Gibco, USA) supplemented with 10% FBS plus 1% streptomycin-penicillin. SCLC cell lines H69, H446, H69R and H446R were obtained from Zhujiang Hospital (Guangzhou, China). Two dual cisplatin (DDP)/epirubicin (EPI) resistant clones, H69R and H446R, were originated from SCLC cell lines, H69 and H446, by treating parental cells with increasing concentration of cisplatin and epirubicin. Cells were maintained in RPMI-1640 medium containing 10% FBS plus 1% streptomycin-penicillin. All cells were incubated at 37°C with 5% CO_2_.

### Patient samples

Clinical breast cancer patient tissue samples were obtained from Guangdong Provincial People's Hospital (Guangzhou, China). 18 Paraffin-embedded lung cancer tissue sections were obtained from Nanfang Hospital, Southern Medical University (Guangzhou, China). Informed consent was obtained from all patients for the analysis of tumor specimens. All human studies were approved by the Medical Ethics Committee of Southern Medical University.

### Paclitaxel‐resistant breast cancer cells construction

MDA-MB-231 cells were incubated with 5 nM paclitaxel (SIGMA, #MKBZ4464V) for 2 days, and then changed with fresh medium until cells recovered. Under gradually increasing concentration of paclitaxel treatment, part of cells still maintained high viability at the maximum concentration of paclitaxel (50 nM). 231R (paclitaxel-resistant) cells were stored for further analyses.

### Stable lentiviral transfection

HEK293T cells were cultured in DMEM supplemented with 10% FBS and 1% streptomycin/penicillin. About 50-60% confluence, HEK293T cells were transfected by PEI transfection reagent (1 mg/ml), using (shRNA-puro plasmids, psPAX2 plasmid, pMD2.G plasmid) packaging system. After 48 and 72 hours respectively, gathering the culture supernatant of HEK293T cells to transfect breast cancer cell lines, supplemented with 0.1% polybrene. 16 hours later, replaced with fresh medium. 72 hours after puromycin treatment, cells were harvested and subjected to analyses by RT-PCR or western blot. For over-expression of CILK1, the CILK1-overexpression plasmid (tagged with GFP) as well as control empty plasmid were transfected as above. Stable clones were maintained with puromycin (1.0 μg/ml).

### CRISPR knockout generation

SgRNAs targeting CILK1 were designed using the online CRISPR design tool (Red CottonTM, Guangzhou, China). Three sgRNA sequences for human CILK1 are as follows: gRNA1 (ACGGGTCCCGACGCGACAGT), gRNA2 (GACGGCGTCTGACGCACCGA), and gRNA3 (AGCCGCTACGGCCCGAAGAC). The pair of oligos for two targeting sites was annealed and ligated to the YKO-LV005 vector (Ubigene Biosciences Co., Ltd., Guangzhou, China). The sgRNAs were transfected into cells with lipofectamine 3000 (Thermo Fisher Scientific) according to manufacturer's instructions. After 48 and 72 hours, lentiviral supernatants were collected and incubated with 231R cells to induce Cas9 expression. The 231R cells were selected with 1 μg/ml puromycin. Then, 231R cells were infected with the CRISPR/Cas9 sgRNA lentiviruses as above and selected with hygromycin at 300 μg /ml for 3 days. Knockout clones were identified by western blot.

### Western blot and immunohistochemistry

For western blot, cells were washed with cold PBS twice and lysed in lysis buffer supplemented with NaF (1:20); PMSF (1:50); Aprotinin (1:1000); Leupeptin (1:1000); Na_3_VO_4_ (1:1000). Protein concentration was determined by BCA kit (KEGEN). Proteins were separated on 10-15% SDS-PAGE and then transferred to PVDF membranes. Membranes were blocked for 1.5 h in 5% milk. Following primary antibodies were used: anti-CILK1 (ab196964, Abcam), anti-pCILK1 (Tyr-159) (#103269, Thermo Fisher Scientific), anti-pCILK1 (Thr-157) (SPC-996, StressMarq), anti-CDK1 (GTX108120, GeneTex), anti-pCDK1 (Thr-161) (ZEN-BIOSCIENCE), anti-caspase-3 and anti-cleaved-caspase-3 (Cell Signaling), anti-ERK1/2 (#4695T, Cell Signaling), anti-pERK1/2 (#4370T, Cell Signaling) and anti-β-actin (Santa Cruz). PVDF membranes were washed six times for 5 min each in PBST and incubated for 1h with secondary antibody (HRP-conjugated anti-rabbit/mouse IgG; Santa Cruz Biotech). All membranes were detected by enhanced chemiluminescence (Millipore). Immunohistochemistry was performed on paraffin-embedded breast or lung tissue sections according to standard procedures. Primary antibodies were as follows: anti-CILK1 antibody (HPA001113, ATLAS ANTIBODIES, 1:500 dilution), anti-phospholate-CILK1 (Tyr-159) (SAB4504612, Sigma, 1:100 dilution), anti-ki67 (ZM-0166, ZSGB-BIO), and anti-cleaved-caspase-3 (9664T, CST, 1:500 dilution). Representative IHC images were captured randomly at ×10 or ×40 magnification using Olympus microscope.

### PCR and real-time PCR

RNA was extracted from cells by RNAiso (Qiagen, #74104), and was reverse transcribed using PrimeScript RT Master Mix kit (Takara, RR036A). Purified DNA was used to perform normal PCR and verified by agarose gel electrophoresis, or was quantified by real-time PCR. For quantitative real-time PCR, DNA was analyzed in triplicate by using the SYBR Green Kit (Takara) according to manufacturer's instructions.

### Cell viability assays

For CCK8 assay, cells were seeded in a density of 1-5× 10^3^ cells per well in 96-well plates overnight. Then the cells were subjected to different treatment for 24 h to 96 h. For MTT assay, the medium was replaced with MTT (5 mg/ml) in complete medium. After 4 hours, discarded the supernatant and added DMSO. The absorbance of formazan was measured at 570 nm using a microplate reader (Synergy Neo2). For clonogenic assay, cells were plated approximately 5000 cells/well in 12-well plates in DMEM medium. After 24 hours, cells were treated in triplicate with vehicle or paclitaxel treatment for 48 h. Then cells were washed with PBS and stained with 0.5% crystal violet. When plates were dried, each well was photographed and then quantified using Image J analysis software.

### Flow cytometric analysis of cell cycle and apoptosis

Analysis of cell cycle was carried out by FACS according to the manufacturer's instructions using Cell Cycle and Analysis Kit (Keygen, KGA512). Cells were fixed with 70 % ethanol overnight at 4 °C, then centrifuged at 1000g for 5 min to remove the ethanol followed by PBS washes. The samples were then treated with RNase A for 30 min at 37 °C, and stained with propidium iodide (PI) then analyzed by the flow cytometer. Apoptosis was measured using the Annexin V-FITC/PI staining kit (Keygen, KGA101) according to the manufacturer's instruction. Briefly, after 48 h or 72 h treatment of drugs, breast cancer cells and lung cancer cells were re-suspended in 500 μl of binding buffer. Annexin V-FITC (5 μl) and PI (5 μl) were added into 100 μl cell suspension containing 1×10^5^ cells. Samples were mixed gently and incubated at room temperature for 15 min, shielded from light. Finally, the percentage of apoptotic cells were measured by flow cytometry (BD Biosciences), and the Flowjo software was used for FACS data analysis.

### Drug affinity responsive target stability (DARTS)

1 × 10^7^ cells were washed with cold PBS for three times, and lysed on ice in M-PER buffer (78503, Thermo Fisher), supplemented with protease inhibitor cocktail (cat.11836153001, Roche) and phosphatase inhibitor cocktail (cat. 4906845001, Roche). Cell lysates were centrifuged at 4°C for 10 min at 18000× g for supernatant collection, then transferred into TNC buffer (50 mM Tris-HCl pH 8.0, 50 mM NaCl and 10 mM CaCl_2_), and protein concentrations were measured by BCA assay. Then the samples were analyzed in triplicate and incubated with 20 μM CILK1-C30/28 or DMSO for 30 min at room temperature and subsequently digested with varying concentrations of pronase (cat.10165921001, Roche) for 5 min. The reaction was then quenched by addition of protease inhibitor cocktail, and the sample was mixed immediately on ice for 15 min. Finally, the samples were subjected to western blot analysis to determine the abundance of CILK1.

### Cellular thermal shift assay (CETSA)

Cells with 80% confluence were treated with 10 μM CILK1-C30/28 or vehicle (DMSO) for 30 min. Cells were harvested and washed with cold PBS, then suspended in 500 μl PBS supplemented with proteinase and phosphatase inhibitor cocktail (100:1). The cells lysates were then subjected to 3 cycles of freeze-thawing between liquid nitrogen and a water bath at 37 °C. The lysates were centrifuged at 4°C for 20 min at 20000× g for supernatant collection, then supernatants were aliquoted into two equal portions and transferred to new EP tubes. Subsequently, 30 μM C28/30 or DMSO was added to the samples and incubated at 37 °C for 30 min using rotarod apparatus. Then the lysates were distributed into 200 μl PCR tubes with 20 μl volume and each tube was designated in a temperature gradient range from 45 to 70°C. Samples were heated at their indicated temperatures for 3 min in a PCR thermal cycler. Immediately after heating, sample tubes were removed and was then allowed to stand at room temperature for 3 min. Lastly, lysate samples were boiled for 5 min at 100°C after the addition of 2× loading buffer, then subjected to western blot analysis. Intensity of CILK1 protein bands were quantified through Image J software. All experiments were performed in triplicate.

### Surface Plasmon Resonance (SPR)

SPR measurement was performed using PlexArray HT A100 instrument. The CILK1 protein was purchased from Abcam (ab101774). CILK1-C30 at different concentrations (1.5625 μM to 25 μM) was run over the SPR instrument with a 3D Dextran chip. The binding and dissociation rates were measured at a flow rate of 25 μL/min. Curves were corrected for nonspecific binding by subtracting the signal obtained for the negative control flow cell. The equilibrium KD was derived by fitting to a 1:1 Langmuir binding model using BIAevaluation data evaluation software.

### TMT quantitative phospho-proteomic analysis

The protein suspensions were digested with 4 μg trypsin (Promega) in 40 μl DS buffer overnight at 37 °C, and the resulting peptides were collected as a filtrate. The peptides of each sample were desalted on C18 Cartridges, concentrated by vacuum centrifugation and reconstituted in 40 µl of 0.1% (v/v) formic acid. The peptide content was estimated by UV light spectral density at 280 nm using an extinctions coefficient of 1.1 of 0.1% (g/l) solution that was calculated on the basis of the frequency of tryptophan and tyrosine in vertebrate proteins. 100 μg peptide mixture of each sample was labeled using iTRAQ reagent according to the manufacturer's instructions (Applied Biosystems). The labeled peptides were mixed, concentrated by a vacuum concentrator and resuspended in 500 μL 1×DHB buffer. Then, TiO2 beads were added and agitated for 2 h. The centrifugation was carried out for 1 min at 5000 g, resulting the beads. And washed with 50 uL of washing buffer I three times and then 50 uL of washing buffer II three times to remove the remaining non-adsorbed material. Finally, the phospho-peptides were eluted with 50 uL of elution buffer three times, followed by lyophilization and MS analysis.

Each fraction was injected for nanoLC-MS/MS analysis. LC-MS/MS analysis was performed on a Q-Exactive mass spectrometer (Thermo Scientific) that was coupled to Easy nLC (Proxeon Biosystems, now Thermo Fisher Scientific) for 120 min. MS data was acquired using a data-dependent top10 method dynamically choosing the most abundant precursor ions from the survey scan (300-1800 m/z) for HCD fragmentation. Automatic gain control (AGC) target was set to 3e6, and maximum inject time to 10 ms. Dynamic exclusion duration was 40.0 s. Survey scans were acquired at a resolution of 70,000 at m/z 200 and resolution for HCD spectra was set to 17,500 at m/z 200, and isolation width was 2 m/z. Normalized collision energy was 30 eV and the underfill ratio, which specifies the minimum percentage of the target value likely to be reached at maximum fill time, was defined as 0.1%. Benjamini-Hochberg correction for multiple testing was further applied to adjust derived p-values. And only functional categories and pathways with p-values under a threshold of 0.05 were considered as significant.

### Kinase assay

Kinase assay was performed in 30 μL reaction. 2 μg recombinant CILK1 protein was incubated with 10 μg ERK1 or ERK2 protein as substrate, and kinase buffer (50 mM HEPES PH 7.5, 10 mM MgCl_2_, 5 mM DTT, 0.5 mM ATP) was added to make up a final volume of 30 μl for 0.5 h at 30 °C. The reaction was terminated by adding 10 μL 4× SDS-PAGE loading buffer and subsequent incubation at 95 °C for 6 min. Then, the proteins were separated on a 10% SDS-PAGE gel and analyzed by western blot using anti-pERK1/2 antibody (1:5000). For mass spectrometry-protein identification, after proteins were separated by SDS-PAGE, the gel was stained with Coomassie brilliant blue G-250. The ERK1/2 bands were excised and digested with trypsin, followed by mass spectrometry analysis. Peptide identification was performed on a Q-Exactive mass spectrometer (Thermo Scientific) that was coupled to Easy nLC (Thermo Fisher Scientific). LC-MS/MS data were analyzed using MaxQuant software version 1.5.3.17 (Max Planck Institute of Biochemistry in Martinsried, Germany) against the UniProtKB databases. Students t-test was performed for each phospho-peptide between the control and kinase group.

### Cell-derived xenograft

Animal experiments were approved by the Institutional Animal Care and Ethical Committee of Southern Medical University. Female Balb/c nude mice were purchased from Guangdong Medical Laboratory Animal Center. The mice were housed under pathogen-free conditions. MDA-MB-231 cells that transfected with lentivirus-based sh-CILK1 or sh-vector plasmids were injected subcutaneously into the mammary fat pads of female Balb/c nude mice (5 weeks old). 1 × 10^6^ MDA-MB-231 cells were suspended in 50 % Matrigel in DMEM medium. The number of mice is as follows: control group (*n* = 7), shCILK1-1 group (*n* = 5) and shCILK1-2 group (*n* = 7). The tumor volume was calculated as follows: (length × width^2^)/2. For in vivo administration experiments, 4-week-old female Balb/c nude mice were transplanted with 1.5 × 10^6^ MDA-MB-231 cells, suspended in 75 μl of Matrigel (1:1 mixture ratio of Matrigel and DMEM medium), orthotopically into the mammary fat pads of mice. When the tumor volume reached approximately 150 mm^3^, the mice were randomly divided into four groups (n=10). Paclitaxel (Selleck, S1150) was given every 72 h at a dose of 10 mg/kg by intraperitoneal (i.p.) injection; CILK1-C30 was given every 48 h at a dose of 10 mg/kg via intraperitoneal (i.p.) injection.

### Patient-derived xenograft

A patient-derived xenograft (PDX) model of TNBC was obtained from Zhujiang Hospital after patient informed consent. Excised tumor tissue was minced into fragments and transplanted into the mammary fat pad of NSG female mice (4-6 weeks). Mice were processed within approximately 3 weeks after tumor implantation. When the tumor size reached 150 to 200 mm^3^, the mice were randomly divided into four groups. Paclitaxel (Selleck, S1150) was given every 72 h at a dose of 10 mg/kg by intraperitoneal (i.p.) injection; CILK1-C30 was given every 48 h at a dose of 10 mg/kg via intraperitoneal (i.p.) injection.

### Statistical analysis

Experiments of immunoblot, apoptosis, cell viability, and cell cycle were performed at least three times. The paired t-test was used for statistical analysis between two groups. Significance was labelled with *p < 0.05, **p < 0.01, ***p < 0.001. Values for measurements were expressed as the mean ± SD unless otherwise noted.

## Results

### CILK1 expression is up-regulated in breast cancer

**Figure 1:**
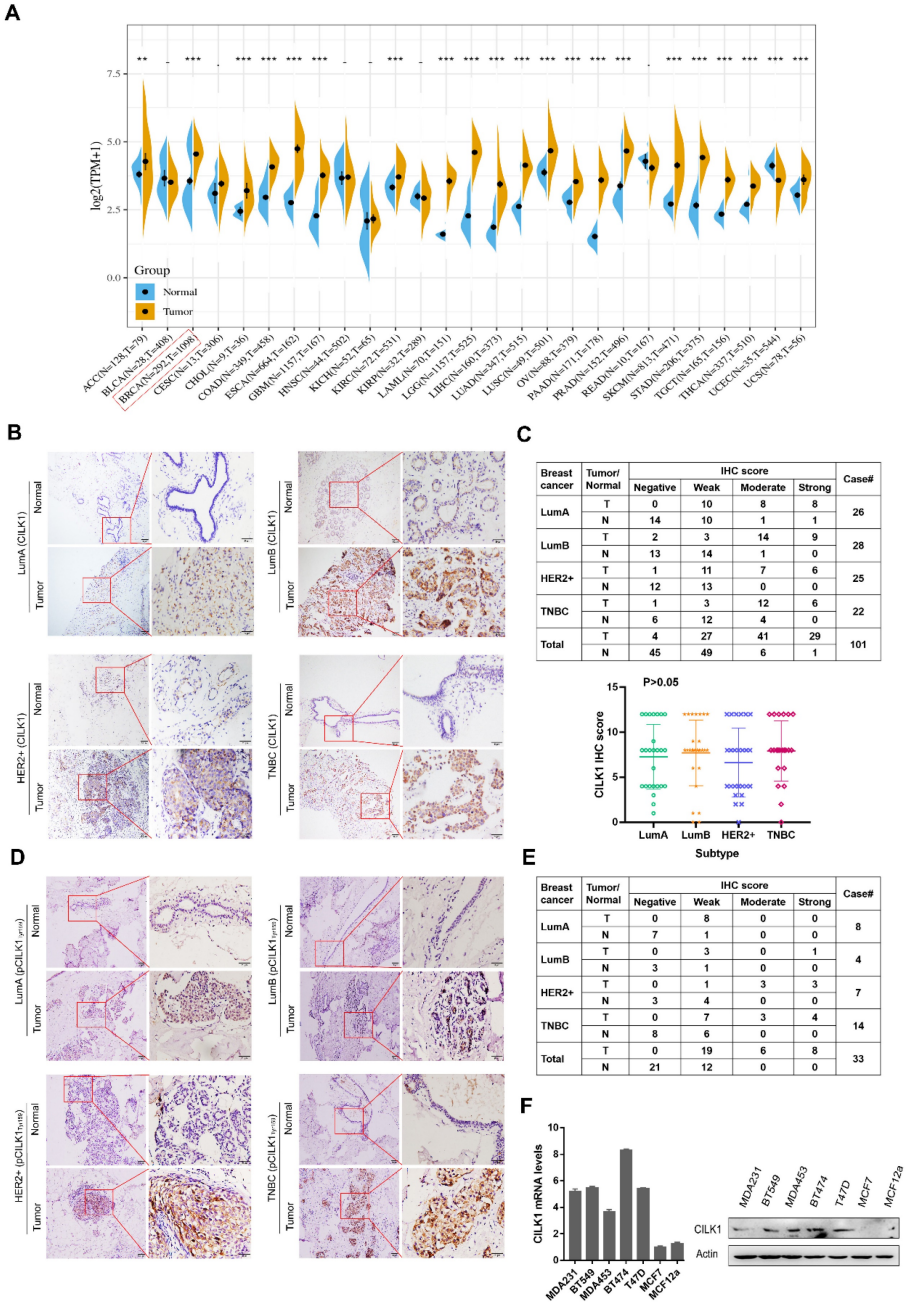
** CILK1 expression is up-regulated in breast cancer.** (**A**) Pan-cancer analysis of CILK1 expression across 27 kinds of human cancer tissues with matched adjacent normal tissues (**p < 0.01, ***p < 0.001). (**B**) IHC staining for CILK1 in 4 subtypes of breast cancer patients (n = 101). Each one typical staining of patient specimen (adjacent normal tissue, *upper*; tumor tissue, *bottom*) from 4 subtypes, respectively, was shown. 10× magnitudes (*left*); 40× magnitudes (*right*). (**C**) The IHC scores of CILK1 in specimens from Lum-A (n = 26), Lum-B (n = 28), HER2^+^ (n = 25), and TNBC (n = 22) subtypes were shown. (**D**) IHC staining for phospho-CILK1 (Tyr-159) in 4 subtypes of breast cancer patients (n = 33). Each one typical staining of patient specimen (adjacent normal tissue, *upper*; tumor tissue, *bottom*) from 4 subtypes, respectively, was shown. 10× magnitudes (*left*); 40× magnitudes (*right*). (**E**) The IHC scores of phospho-CILK1 (Tyr-159) in specimens from Lum-A (n = 8), Lum-B (n = 4), HER2^+^ (n = 7), and TNBC (n = 14) subtypes were shown. (**F**) CILK1 mRNA (*left*) and protein (*right*) levels were detected in a panel of breast cell lines by quantitative real-time PCR and western blot.

By analysis of The Cancer Genome Atlas (TCGA) and Genotype-Tissue Expression (GTEx) databases, we observed an increased mRNA expression of CILK1 in a variety of human cancers including breast cancer, lung cancer, esophageal cancer and liver cancer* etc.* (Fig. 1A). To investigate its protein expression status in breast cancer, we collected 101 cases of post-operative clinical paired breast cancer specimens for immunohistochemical analysis. Our data showed that the expression of CILK1 in four major breast cancer subtypes including Luminal A, Luminal B, HER2^+^ and Triple-negative, was significantly higher than that of adjacent normal breast tissues (Fig. 1B, S1A). CILK1 was positively expressed in almost all breast cancer specimens (96%), while 45% normal breast tissues were negative staining. The IHC score of 29% tumor specimens were strong, whereas only 1% normal tissues exhibited strong signals. Interestingly, CILK1 protein expression was not significantly linked to specific breast cancer subtype (Fig. 1C). And, there was no apparent difference of CILK1 mRNA level among breast cancer subtypes based on *Gene Expression* Omnibus (GEO) databases (Fig. S1B).

Similar to the TXY motif of MAP Kinases, CILK1 protein contains a corresponding TDY motif. Phosphorylation of Tyr-159 residue in the TDY motif confers CILK1 with the basic catalytic activity 18. To further observe the clinical relevance of active CILK1 protein, the expression status of phosphorylated CILK1 (Tyr-159) was analyzed in tissue specimens from 33 breast cancer patients with high CILK1 protein level. The results showed that the accumulation of phospho-CILK1 was also significantly up-regulated in breast tumor tissues, compared with the adjacent non-cancerous tissues (Fig. 1D, S1C). Furthermore, 100% of breast cancer cases expressed phospho-CILK1, suggesting that the kinase activity of CILK1 might be required for breast cancer tumorigenicity (Fig. 1E). Subsequently, the analysis of CILK1 expression in a panel of cultured breast cell lines indicated that it has much lower mRNA and protein levels in luminal A breast cancer cell line (MCF7) and non-tumorigenic breast cell line (MCF12a) (Fig. 1F).

Collectively, these data indicate that the expression of CILK1 is up-regulated in breast cancer, suggesting that it might play a role in breast cancer.

### CILK1 is critical for breast cancer cell proliferation

**Figure 2:**
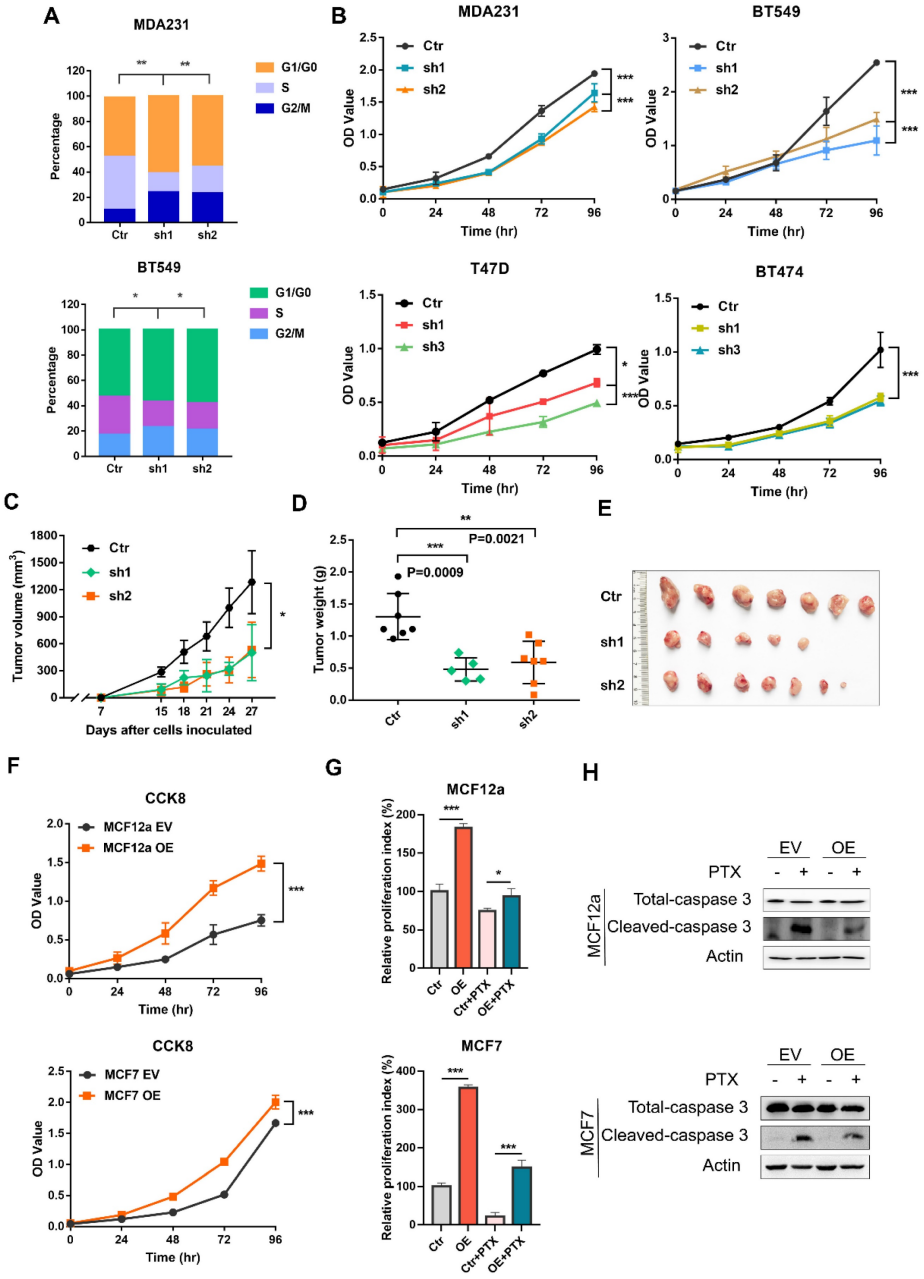
** CILK1 is critical for breast cancer cell proliferation.** (**A**) Quantification of cell cycle percentage from flow cytometry analyses in MDA-MB-231 (*upper*) and BT549 (*bottom*) cells. (**B**) CCK8 assay was performed in vector control and CILK1-knockdown MDA-MB-231, BT549, T47D and BT474 cells. (**C**) Xenograft tumor growth curves were shown, mice were injected with control MDA-MB-231 cells (n=7) or CILK1-knockdown clones (sh-1, n = 5 and sh-2, n = 7). (**D**) Tumor weight was shown. (**E**) The photo of tumors was shown. (**F**) CCK8 assay was done to determine the effect of CILK1-overexpression on the proliferation of MCF12a (*upper*) and MCF7 (*bottom*) cells. (**G**) Relative cell proliferation index in DMSO- or paclitaxel-treated control or CILK1-overexpression cells was calculated based on crystal violet staining. (**H**) Caspase-3 and cleaved-caspase-3 levels were detected in MCF12a and MCF7 cells after treated with paclitaxel (5 nM) or DMSO for 48 hours. Error bars represent mean ± SD, 3 independent experiments in triplicate were performed, data were analyzed by unpaired Student t-test in Prism. *p < 0.05, **p < 0.01, ***p < 0.001.

It remains completely unclear whether CILK1 is required for tumor cell proliferation. We used a panel of breast cancer cell lines, including MDA-MB-231 and BT-549 (Triple-negative), T47D (Luminal) and BT-474 (HER2^+^) to construct stable CILK1-knockdown clones (Fig. S2A). Flow cytometry analysis showed that the proportion of G1 phase in CILK1-knockdown clones greatly increased versus control, while the proportion of S phase decreased (Fig. 2A). Meanwhile, knockdown of CILK1 significantly slowed down the proliferation rate of all four breast cancer cell lines (Fig. 2B). To certify its biological function, wild-type and CILK1-silencing MDA-MB-231 cells were implanted in the mammary fat pad of nude mice. Results showed that silencing of CILK1 robustly inhibited the tumor cell growth potential (Fig. 2C), and significantly reduced the size and weight of tumors in comparison with the vector control group (Fig. 2D-E, S2B). Conversely, we constructed stable CILK1 over-expression clones in MCF12a and MCF-7 cells (Fig. S2C). The over-expression of *CILK1* gene markedly enhanced the proliferation rate of these two cell lines (Fig. 2F, S2D). Following, we sought to observe whether CILK1 exerts any effect on cell viability. Because paclitaxel exhibits strong cytotoxicity and is frequently used to induce cell apoptosis 19, we then tried to see whether the up-regulated CILK1 is able to counteract the paclitaxel-mediated cytotoxicity. The results revealed that over-expression of this gene apparently alleviated paclitaxel-mediated decrease of cell viability (Fig. 2G), and suppressed the pro-apoptotic caspase-3 activation (Fig. 2H). These findings indicate that CILK1 is required for breast cancer cell proliferation.

### Elevated CILK1 expression confers resistance to chemotherapy

**Figure 3:**
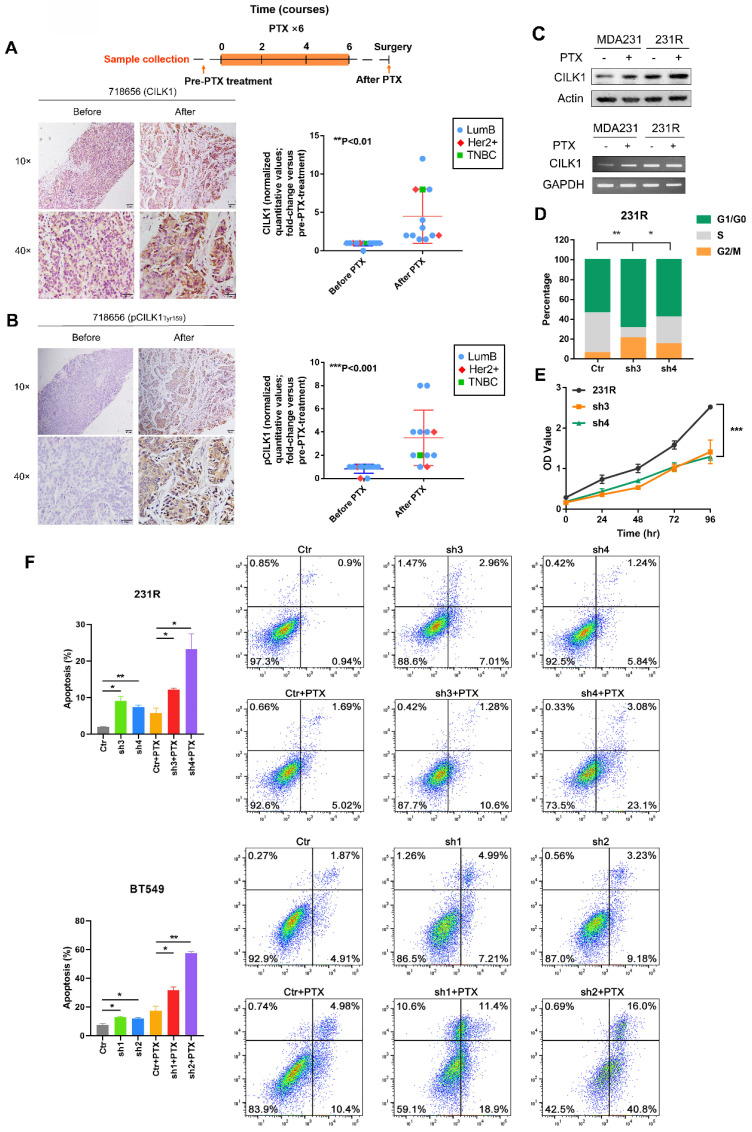
** Elevated CILK1 expression confers resistance to chemotherapy.** (**A**) Scheme of the paclitaxel treatment timeline and time-point of tumor tissue collection in clinical breast cancer patients (*top*). IHC staining of CILK1 before and after paclitaxel treatment in one typical breast cancer patient (*left*); IHC-based quantification of CILK1 expression in tumor tissues of breast cancer patients (n = 12) before and after PTX treatment (*right*). PTX: Paclitaxel. (**B**) IHC staining of phospho-CILK1 (Tyr-159) before and after paclitaxel treatment in one typical breast cancer patients (*left*); IHC-based quantification of phospho-CILK1 (Tyr-159) expression in tumor tissues of breast cancer patients (n = 12) before and after PTX treatment (*right*). (**C**) CILK1 levels were analyzed in MDA-MB-231 and 231R cells by western blot (*upper*) and PCR (*bottom*) in response to paclitaxel (10 nM) or DMSO treatment for 24 hours. (**D**) Quantification of cell cycle percentage in control and CILK1-silencing 231R cells by flow cytometry. (**E**) CCK8 assay was performed to determine the effect of shRNA-mediated CILK1-knockdown on the proliferation of 231R cells. (**F**) Annexin V-FITC flow cytometry assay was used to measure the pro-apoptotic effect of paclitaxel in vector control and CILK1-silencing 231R and BT549 cells. Error bars represent mean ± SD, and the experiments were repeated three times. A representative experiment was shown, *p < 0.05, **p < 0.01, ***p < 0.001.

Chemotherapy is important for clinical treatment of breast cancer, especially for Triple-negative subtype patients. Since enhanced CILK1 expression impairs the cytotoxic effect of paclitaxel, we sought to comprehensively study whether CILK1 is involved in the chemotherapy resistance. To investigate the clinic-pathological relevance of CILK1 in drug resistance, we detected the expression of phospho-CILK1 (Tyr-159) and total-CILK1 protein by IHC in a cohort of clinical breast cancer patients. The patient cohort is consisted of 12 breast cancer cases that treated with a docetaxel-based neoadjuvant chemotherapy of 6-8 courses (average of three weeks per course), including 9 luminal B, 2 HER2^+^ and 1 TNBC patients. We compared them with matching biopsy samples which were taken before neoadjuvant chemotherapy to determine whether phospho- or total CILK1 levels have significant alterations. Apparently, we found a significant increase of CILK1 expression in the samples that collected after docetaxel-based neoadjuvant chemotherapy (Fig. 3A, S3A). In particular, we observed that the phospho-CILK1 (Tyr-159) level was also strikingly elevated in the after-neoadjuvant-chemotherapeutic samples compared with the before cohort, strongly suggesting that kinase-active CILK1 might participate in the chemoresistance (Fig. 3B, S3B).

To mimic the chemotherapy resistance, we generated MDA-MB-231-derived paclitaxel-resistant 231R cells by treatment with increasing concentration of paclitaxel gradually. Intriguingly, significant up-regulation of CILK1 expression in 231R cells was observed compared with the parental cells in the presence or absence of paclitaxel treatment (Fig. 3C). In addition, we found the cell lines (MDA-MB-231 and BT549) with high CILK1 levels exhibit more resistance to paclitaxel compared to MCF12a and MCF7 cells (Fig. S4A). To study whether the up-regulated CILK1 is responsible for the formation of paclitaxel resistance phenotype, we silenced the CILK1 expression in 231R cells using both shRNA and Crispr/Cas9 ways (Fig. S4B). Consistently, silencing of CILK1 induced G1 phase growth arrest (Fig. 3D). Also, CILK1-silencing itself strongly suppressed the growth of 231R cells (Fig. 3E, S4C). We next investigated whether CILK1 affects the drug sensitivity of paclitaxel. The data showed that the down-regulation of CILK1 promoted the killing effect of paclitaxel in breast cancer cells, as well as in the paclitaxel-resistant cells (Fig. 3F, S4D). Meanwhile, CILK1-knockdown also potentiated the pro-apoptotic caspase-3 activation (Fig. S4E). Taken together, these data indicate that the elevated CILK1 not only facilitates the proliferation of breast cancer cells, also engages in the formation and maintenance of paclitaxel-resistance phenotype.

### Identification of selective inhibitors of CILK1

**Figure 4:**
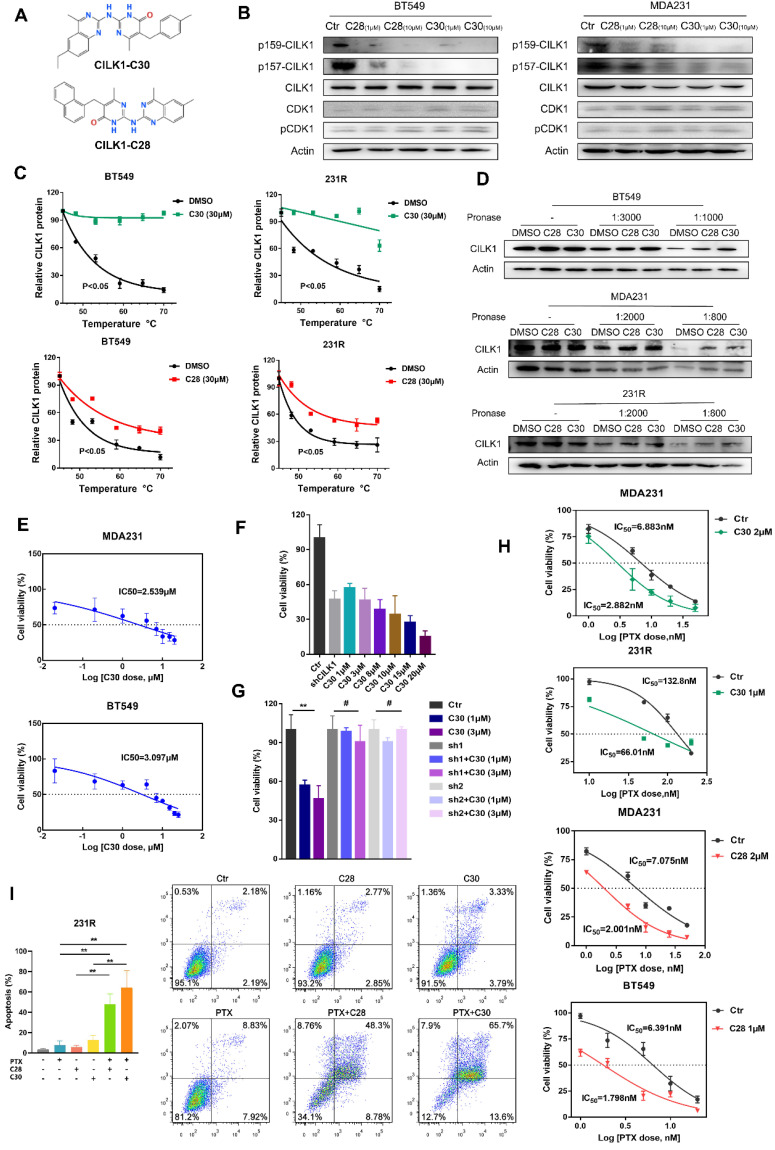
** Identification of selective inhibitors of CILK1.** (**A**) The chemical structure formula of CILK1-C30/C28. (**B**) Western blots were performed to detect the phosphorylation of CILK1 and CDK1 in two breast cancer cell lines, which treated with DMSO or CILK1-C28 or CILK1-C30 for 24 h. (**C**) Melt curves of CILK1 protein in CETSA analysis of BT549 and 231R cells that treated with CILK1-C30 (*upper*) or CILK1-C28 (*bottom*) or DMSO. The graph showed the quantification of CILK1 protein at different temperature points based on western blot analyses. (**D**) Cancer cell lysates treated with CILK1-C28 (10 μM) or CILK1-C30 (10 μM) was subjected to DARTS assay with pronase treatment. Lysates treated with DMSO served as negative control. (**E**) The inhibitory concentration curves of MDA-MB-231 and BT549 cells that treated with CILK1-C30 were determined by CCK8 assay, and the IC_50_ values were calculated using GraphPad Prism. (**F**) CCK8 assay was done in control or CILK1-knockdown MDA-MB-231 cells, and various concentrations of CILK1-C30 were used to treat control MDA-MB-231 cells. (**G**) CCK8 assay was done in control or CILK1-knockdown MDA-MB-231 cells, treated with vehicle or CILK1-C30 (1 uM or 3 μM) for 48 h. Data are presented as mean ± SD; **p < 0.01, #p > 0.05; one-way ANOVA. (**H**) Inhibitory IC_50_ values of paclitaxel were measured by CCK8 assay in cancer cells that pre-treated with CILK1-C30 or C28. (**I**) Flow cytometry was performed to measure the pro-apoptotic effect of CILK1-C30/28 combined with paclitaxel in 231R cells. Error bars represent mean ± SD, 3 independent experiments in triplicate were performed, data were analyzed by unpaired Student t-test in Prism. *p < 0.05, **p < 0.01, ***p < 0.001.

Next, we sought to develop the specific inhibitors of CILK1. The full activation of CILK1 requires dual Tyr-159/Thr-157 phosphorylation of the TDY motif 20. Initial auto-phosphorylation of Tyr-159 only confers it with basal kinase activity, the full activation requires additional phosphorylation of Thr-157. Moreover, CILK1 is a Cyclin dependent kinase 1 (CDK1)-related kinase with similar N-terminal catalytic domains 2, 9. Based on the known structure of CDK1 protein and high sequential similarity between CILK1 and CDK1, we constructed the 3D protein structure of kinase domain of CILK1 (Y4-F284) by homology modelling. Through MOE Site Finder, we found that near the proton transfer active center and the ATP binding region, there is a binding pocket in the protein that is suitable for binding of small-molecule compounds. The pocket can hold the molecules with up to 229 atoms, has enough space to interact with most of small-molecule compounds. The pocket contains 79 amino acids with strong hydrophobicity including Tyr-159/Thr-157 residues. Tentative compounds may be able to occupy the pocket and block the dual phosphorylation. Based on Chemdiv library, we screened the compounds which have high affinity to CILK1 and relatively low affinity to CDK1 protein, by virtual screening and molecular docking. Following, tentative compounds were tested for their inhibitory activity towards Tyr-159/Thr-157 phosphorylated CILK1. Considering the potential off-target effects, we also assessed whether they can alter Thr-161 phosphorylation of CDK1. Among candidates, two compounds, CILK1-C28 and CILK1-C30 (Fig. 4A), showed potent inhibitory effect against the Tyr-159/Thr-157 phosphorylation of CILK1, but not against the Thr-161 phosphorylation of CDK1 (Fig. 4B and S5A), revealing that they specifically inhibit the phosphorylation of CILK1.

Next, two compounds were tested for their ability to bind and stabilize CILK1 protein by Cellular Thermal Shift Assays (CETSA). CETSA is based on the biophysical principle of ligand-induced thermal protein stabilization for assessing target engagement 21. The test was performed in MDA-MB-231, BT-549 and 231R cells, which showed an obvious increase in the thermal stability of CILK1 upon the addition of CILK1-C28/30 (Fig. 4C, S5B-C). To further examine the target engagement of two chemicals and determine the binding affinity, we carried out Drug Affinity Responsive Target Stability (DARTS) assay. DARTS is a straightforward approach for small molecules to identify potential protein targets 22. As expected, dose-dependent attenuation of signals about CILK1 protein stability was observed in vehicle-treated controls, whereas CILK1 protein became protease-resistant in the presence of CILK1-C28/30 (Fig. 4D), indicating that these two compounds indeed bind to CILK1 protein. We next applied surface plasmon resonance (SPR) analysis to investigate whether CILK1-C30 directly interacts with CILK1. The biochemical parameters for the binding of CILK1-C30 and CILK1 were measured. Our results showed that the equilibrium dissociation constant (KD) of CILK1-C30 toward CILK1 is 0.108 μM (Fig. S5D). Collectively, above results indicate that two compounds directly recognize and bind to CILK1 protein.

We proceeded to observe whether two chemicals have anti-proliferation effect, and obtained the cell-growth inhibitory IC_50_ values of CILK1-C30 and CILK1-C28 respectively, in MDA-MB-231 (2.539 μM and 3.313 μM) and BT-549 (3.097 μM and 2.938 μM) (Fig. 4E, S5E). In addition, we compared the cell-growth-inhibition efficacy between CILK1-knockdown and small molecule-mediated inhibition, and observed that cell viability gradually decreased under the treatment of two chemicals with increasing concentrations, the incubation of 3 μM CILK1-C30/28 resulted in a similar inhibitory effect as that of CILK1-knockdown (Fig. 4F, S6A). In order to determine whether the inhibitory effects of CILK1-C30/28 on breast cancer cell proliferation rely on the repression of CILK1, cell viability assay was performed in vector control and CILK1-knockdown cells. The results revealed a significant decrease of viability in control group upon CILK1-C30/28 treatment, whereas there were no obvious differences in CILK1-silencing cells in the presence or absence of two compounds (Fig. 4G, S6B). These data confirm the specificity of two chemicals on the inhibition of CILK1.

We kept on investigating the combinatorial effect of paclitaxel and CILK1-C30/28 on breast cancer cells. Measurements showed that the pre-treatment of two compounds was able to strongly decrease the inhibitory IC_50_ values of paclitaxel and sensitize breast cancer cells to chemotherapy (Fig. 4H, S6C).

Annexin-V based flow cytometry assay demonstrated that the treatment with CILK1-C30 or CILK1-C28 alone significantly induced apoptosis in MDA-MB-231 and BT-549 cells, the combination of CILK1-C30/28 with paclitaxel produced more greater toxicity in tumor cells (Fig. S6D). Similarly, the incubation with CILK1-C30/28 plus paclitaxel triggered vigorous apoptosis in 231R derivatives (Fig. 4I). Altogether, these data support the contention that selective CILK1 inhibitors exhibit strong cytotoxicity to tumor cells and are able to potentiate the pro-apoptosis effect of chemotherapeutic reagents.

### CILK1 directly phosphorylates ERK1

**Figure 5:**
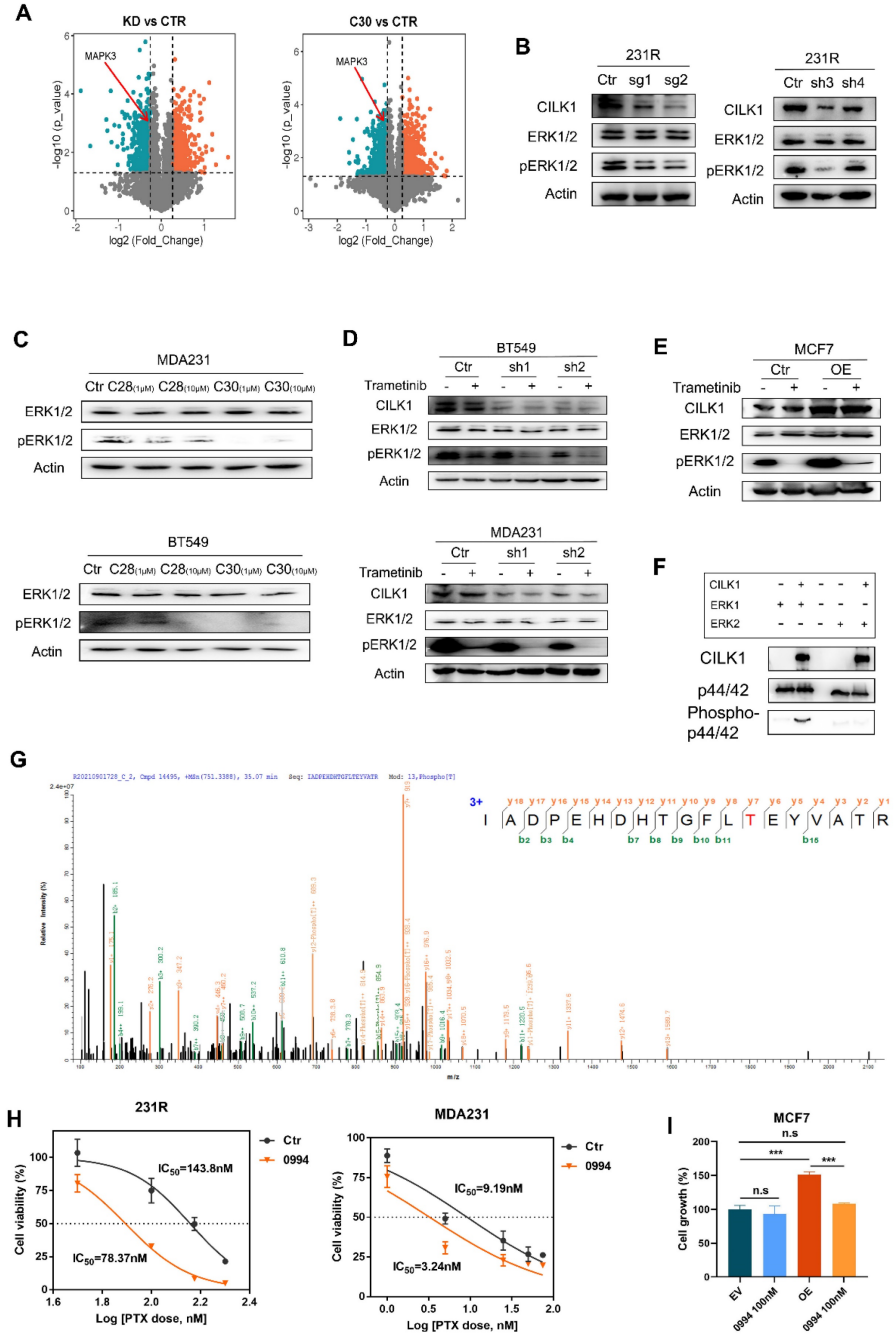
** CILK1 directly phosphorylates ERK1.** (**A**) Volcano plots of the quantitative TMT‐based proteomic analysis, which indicated MAPK3 as one of the differential phospho-proteins. The data of CILK1-knockdown or CILK1-C30-treatment were shown respectively. Blue and red dots represented significantly down- and up-regulated phospho-proteins (p < 0.05). (**B**) Western blot was performed to detect the expression levels of ERK1/2 and phospho-ERK1/2 in sgRNA (*left*) or shRNA (*right*)-mediated CILK1-knockdown 231R cells. (**C**) Detection of the expression levels of ERK1/2 and phospho-ERK1/2 in CILK1-C28/30 treated cells. (**D**) Western blot was done to detect the expression levels of CILK1, ERK1/2 and phospho-ERK1/2 in control and CILK1-knockdown BT549 and MDA-MB-231 cells, which treated with vehicle or trametinib (10 nM) for 30 min. (**E**) Western blot was performed to detect the expression levels of CILK1, ERK1/2 and phospho-ERK1/2 in control and CILK1-overexpression MCF7 cells, which treated with vehicle or trametinib (10 nM) for 30 min. (**F**) CILK1 kinase assay was done using purified ERK1 or ERK2 protein as substrate. (**G**) Phosphorylation site of ERK1 protein catalyzed by CILK1 was detected by mass spectrum. (**H**) Inhibitory IC_50_ values of paclitaxel were measured by CCK8 assay in cells pre-treated with GDC-0994 (100 nM) for 48 h, calculated by GraphPad. (**I**) CCK8 assay was done in vector control or CILK1-overexpression MCF7 cells, treated with DMSO or GDC-0994 for 72 h. Statistical data (mean ± SD) were shown. Error bars represent mean ± SD, 3 independent experiments in triplicate were performed, data were analyzed by unpaired Student t-test in Prism. *p < 0.05, **p < 0.01, ***p < 0.001.

To reveal the downstream signals triggered by CILK1, we identified the differential phospho-proteins in CILK1-silencing or CILK1-C30-treated cells through phospho-proteomic analysis by the combination of tandem mass tags (TMT) and TiO2-based phosphor-peptide enrichment followed by liquid chromatography tandem mass spectrometry (LC-MS/MS) analysis (Fig. S7A-B). Especially, the phosphorylation of ERK1 (MAPK3) (Thr-202) and ERK2 (MAPK1) (Thr-185) was obviously decreased upon pharmacologic inhibition and genetic silencing of CILK1 (Fig. 5A, S7C).

ERK1/2 are serine/threonine protein kinases that participate in the Ras/Raf/MEK/ERK signal cascade. This cascade regulates a large variety of processes including cell cycle progression, migration, survival, and differentiation 23, 24. MEK1/2 catalyze the phosphorylation of ERK1/2 proteins at Tyr-204/187 and Thr-202/185 residues. Although ERK1/2 catalyze the phosphorylation of hundreds of substrates, MEK1/2 have narrow substrate specificity. To verify the preliminary results from mass spectrometry, we knocked down *CILK1* gene and revealed the consequent decrease of ERK1/2 phosphorylation (Fig. 5B). Also, the incubation of CILK1-C30/28 resulted in appreciable repression of ERK1/2 phosphorylation, compared with the vehicle controls (Fig. 5C, S7D). To further explore the relationship between MEK1/2 and CILK1 on ERK1/2 phosphorylation, we treated Trametinib, an approved MEK1/2 inhibitor, in vector control and CILK1-knockdown cells, and observed a more profound inhibition of ERK1/2 phosphorylation in the Trametinib-treated/CILK1-silencing cells compared with other three groups (Fig. 5D, S7E). Conversely, we incubated vehicle control or Trametinib in vector control or CILK1-overexpressing cells, the over-expression of CILK1 itself obviously stimulated ERK1/2 phosphorylation, in addition, over-expression of CILK1 partially rescued the down-regulation of ERK1/2 phosphorylation due to Trametinib treatment (Fig. 5E). These data indicate that CILK1 cooperates with MEK1/2 to induce and maintain the phosphorylation status of ERK1/2.

In order to determine whether CILK1 is able to directly catalyze the ERK1/2 phosphorylation, we established a kinase assay with active recombinant CILK1 protein using human ERK1 or ERK2 proteins as substrate, respectively. Interestingly, we found that the addition of active CILK1 protein selectively catalyzed ERK1 phosphorylation, but not ERK2, suggesting that only ERK1 is the direct substrate of CILK1 (Fig. 5F). To further verify the phosphorylation sites in ERK1, protein identification was carried out by QE mass spectrometry analysis. Concordantly, we found that ERK1 protein was phosphorylated at Thr-202 (Fig. 5G). Although CILK1 protein has basal tyrosine auto-phosphorylation activity, we did not observe the Tyr-204 phosphorylation on ERK1 protein in our mass spectrometry analysis.

Targeting Ras/MAPK cascade has been shown promising prospective for cancer therapy 25. GDC‐0994 is a small-molecule inhibitor selective for ERK1/2 kinase activity 26. To investigate the functional role of ERK1 in therapy resistance, we estimated the IC_50_ of paclitaxel in the absence or presence of GDC-0994. The data showed that the IC_50_ values markedly decreased in breast cancer cells and 231R cells after the co-treatment with GDC-0994 (Fig. 5H, S7F). To determine the functional dependence of CILK1 on ERK1, we treated GDC-0994 in vector control or CILK1-overexpressing MCF7 cells, and found that the growth advantage offered by CILK1 expression was completely offset by the addition of GDC-0994, indicating that the oncogenic role of CILK1 is dependent on ERK1 activation (Fig. 5I). Collectively, these results confirm that CILK1 acts as an upstream kinase of ERK1, CILK1 assists Ras/MAPK signaling through activation of ERK1.

### Pharmacological inhibition of CILK1 in TNBC mice models

**Figure 6:**
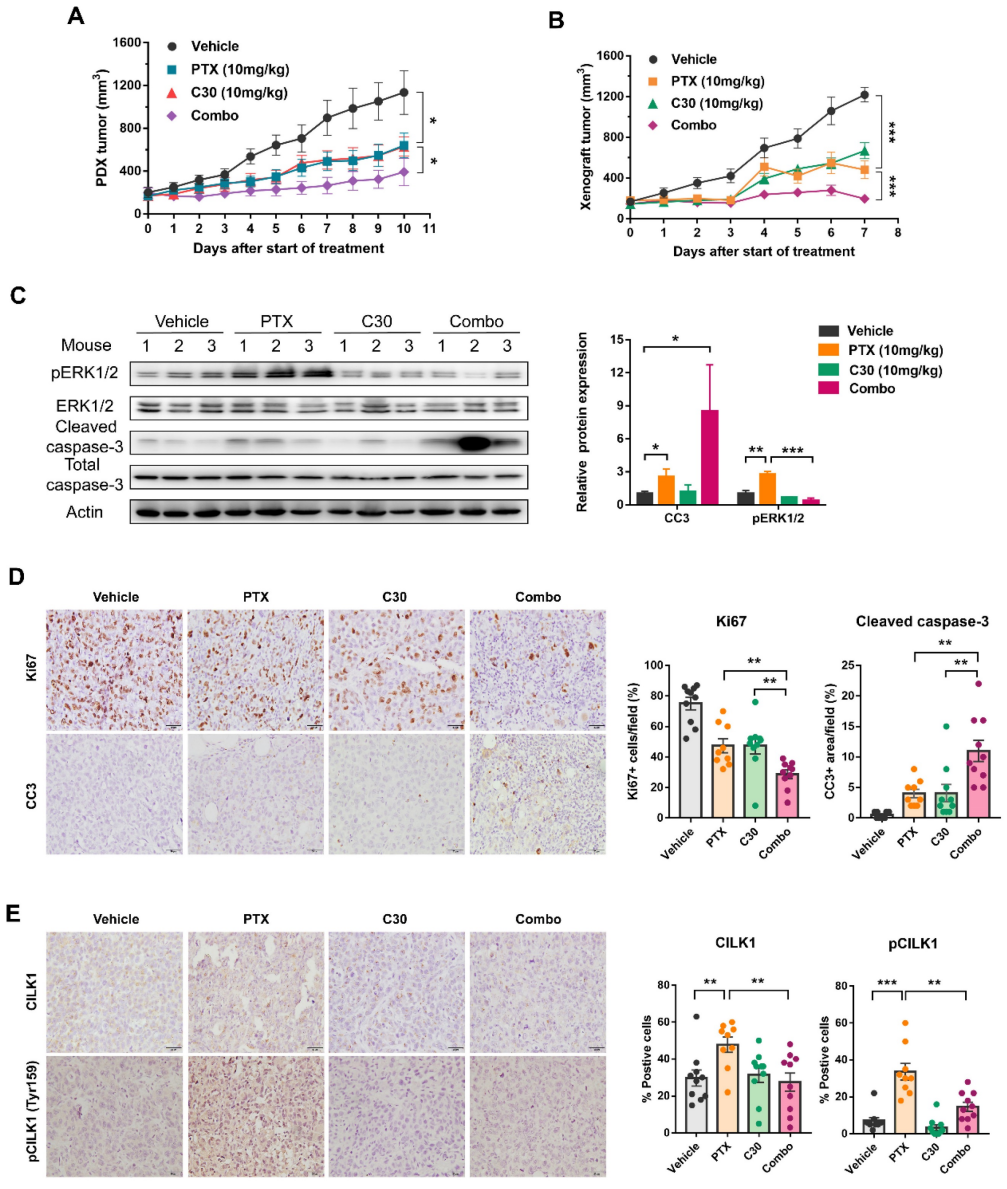
** Pharmacological inhibition of CILK1 in TNBC mice models.** (**A**) NSG mice bearing PDX tumors were administered with CILK1-C30 (10 mg/kg, i.p) every two days, for a total of five injections; or administered with paclitaxel (10 mg/kg, i.p) every three days, for a total of four injections; or administered (i.p) with vehicle control. Growth curves of PDX tumors during 11 days of treatment with vehicle control, or paclitaxel (PTX), or CILK1-C30 as well as PTX/CILK1-C30 combination were shown. (**B**) Balb/c mice bearing MDA-MB-231-derived tumors were administered with CILK1-C30 (10 mg/kg, i.p) every two days, for a total of four injections; or administered with paclitaxel (10 mg/kg, i.p) every three days, for a total of three injections; or administered (i.p) with vehicle control. Growth curves of xenograft tumors during 8 days of treatment with vehicle control, or paclitaxel (PTX), or CILK1-C30 as well as PTX/CILK1-C30 combination were shown. (**C**) Immunoblot analysis and quantification of the indicated proteins from MDA-MB-231-derived tumors. Quantification of proteins was presented in normalized against Actin. Data are presented as mean ± SD. *p < 0.05, **p < 0.01, ***p < 0.001. (**D**) Representative images and quantification of the IHC staining of Ki-67 and cleaved caspase-3 (CC3) in MDA-MB-231-derived tumors. Data are presented as mean ± SD. **p < 0.01 by t-test. (**E**) Quantification and representative immunohistochemistry images of the indicated proteins in MDA-MB-231-derived tumors. Scale bar represents 40 μm. Results are mean ± SD of at least nine mice per group. *p < 0.05, **p < 0.01, ***p < 0.001.

We utilized a TNBC patient-derived xenograft (PDX) mice model to assess whether paclitaxel/CILK1-C30 combination could be a potential therapy in clinical setting. The PDX tumors were treated with paclitaxel at 10 mg/kg/day every 3 days, followed by CILK1-C30 treatment at 10 mg/day every 2 days, for a total treatment of 11 days. As shown, single CILK1-C30 or paclitaxel treatment partially slowed down the tumor growth, the combined treatment led to more robust inhibition of tumor growth (Fig. 6A and S8A). MDA-MB-231-derived xenograft model was also established to test the therapeutic efficacy of the combination of CILK1-C30 with chemotherapy. The xenograft tumors were treated with paclitaxel at 10 mg/kg/day every 3 days, followed by CILK1-C30 treatment at 10 mg/day every 2 days, for a total treatment of 8 days. Of note, single CILK1-C30 or paclitaxel treatment exhibited obvious tumor-suppressive effect, the delay of tumor growth under the united treatment was more pronounced than either single-agent-treated group (Fig. 6B and S8B). The combination of CILK1-C30 and paclitaxel treatment presented a more effective tumor growth inhibition, without significant effect on the body weight of mice (Fig. S8C-D). Phospho-ERK1/2 levels increased upon paclitaxel treatment but were strongly suppressed by the united therapy in these MDA-MB-231-derived tumors (Fig. 6C). United treatment also significantly decreased the proliferation potential (Ki67+ cells) compared to single treatment, and resulted in increasing apoptosis (Cleaved Caspase-3+ cells) in MDA-MB-231-derived tumors (Fig. 6D). We also found the rapid accumulation of total and phosphorylated CILK1 protein following paclitaxel treatment, the combined treatment completely inhibited the expression (Fig. 6E). Together, these data support CILK1 as a promising therapeutic target for TNBC.

### CILK1 is required for chemoresistance of SCLC

**Figure 7:**
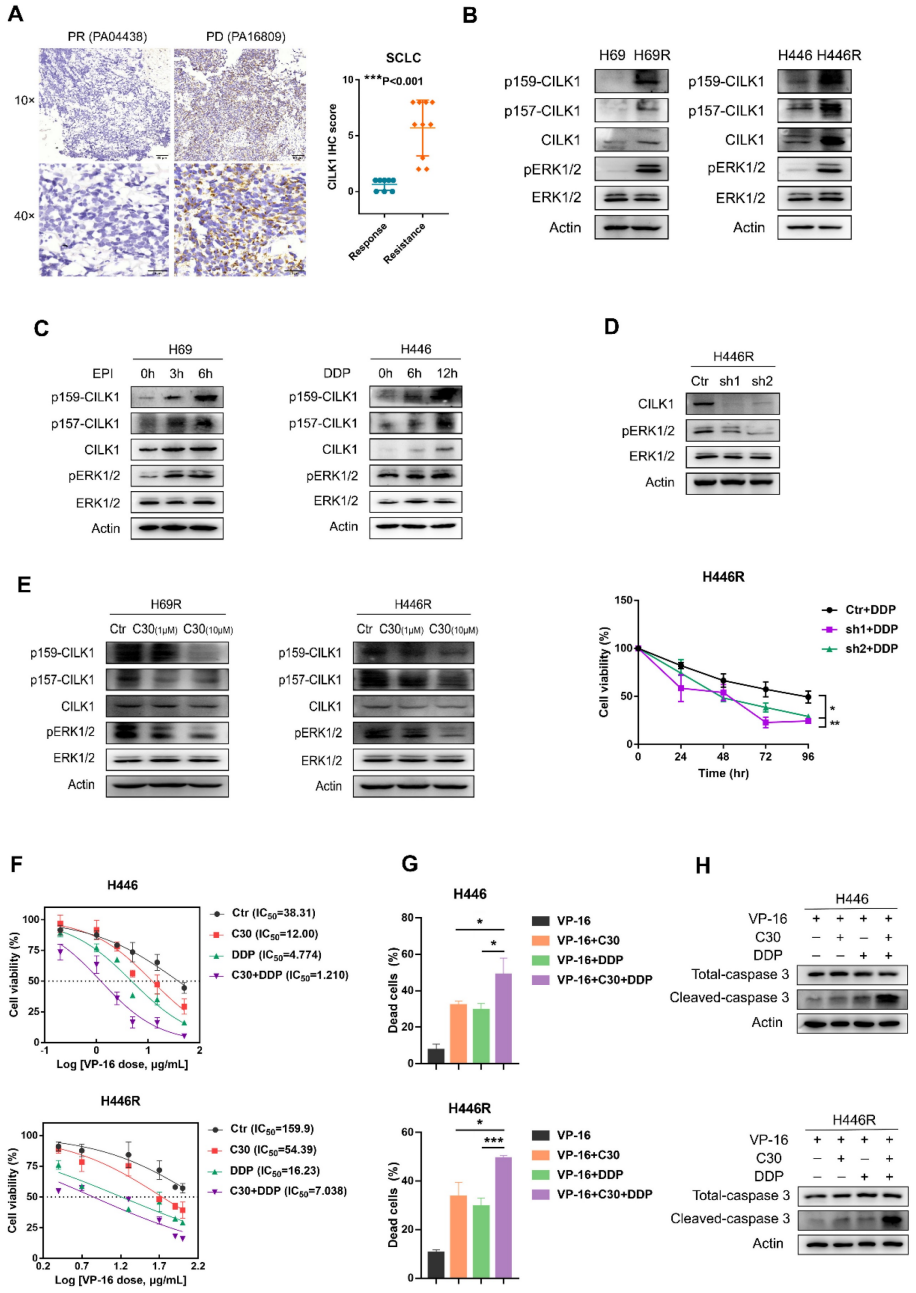
** CILK1 is required for chemoresistance of SCLC.** (**A**) IHC staining and scoring of CILK1 expression in 18 SCLC patients with platinum/etoposide chemotherapy. Sensitive tumors (PR, n = 8) were indicated in blue dots, and resistant tumors (PD or SD, n = 10) were indicated in orange dots. Representative IHC staining of sensitive (*left*) and resistant (*right*) tumor was shown. (**B**) Western blot was performed to detect the phosphorylation of CILK1 and ERK1/2 in H69, H446 and their resistant clones. (**C**) Western blot was performed to detect the phosphorylation of CILK1 and ERK1/2 in H69 and H446 cells, which treated with vehicle or epirubicin (EPI) (0.5 μg/ml) or cisplatin (DDP) (0.5 μg/ml) in indicated time points. (**D**) (*upper*) Western blot was performed to detect the expression levels of CILK1 and phospho-ERK1/2 in control and CILK1-knockdown clones of H446R cells. (*bottom*) CCK8 assay was performed to determine the proliferation of control and CILK1-knockdown H446R cells, treated with cisplatin. (**E**) Western blot was performed to detect the phosphorylation of CILK1 and ERK1/2 in H69R and H446R cells, which treated with vehicle or CILK1-C30 (1 μM and 10 μM) for 24 h. (**F**) IC_50_ values of H446/H446R cells for VP-16 were measured by CCK8 assay, after treatment with C30 (1 μM) or DDP (0.5 μg/ml for H446 and 1.5 μg/ml for H446R) or Combo for 48 h. (**G**) Trypan blue staining was used to detect the percentages of dead cells after treated with indicated agents for 48 hours. Concentrations of agents were: VP-16 (3.5 μg/ml for H446, 30 μg/ml for H446R), C30 (1 μM), DDP (0.5 μg/ml for H446, 1.5 μg/ml for H446R). (**H**) Detection of Caspase-3 and cleaved-caspase-3 in H446 and H446R cells after treated with indicated agents for 24 hours. Concentrations of agents were: VP-16 (5 μg/ml for H446, 40 μg/ml for H446R), C30 (1 μM), DDP (0.5 μg/ml for H446, 1.5 μg/ml for H446R). Error bars represent mean ± SD, 3 independent experiments in triplicate were performed, data were analyzed by unpaired Student t-test in Prism. *p < 0.05, **p < 0.01, ***p < 0.001.

Previous studies implicated a role of CILK1 in lung development and Endocrine-cerebro-osteodysplasia (ECO) syndrome 27, 28. We found the gene expression of *CILK1* was up-regulated in lung cancer versus normal lung tissues by bio-informatic analyses (Fig. 1A). To discern the relevance of CILK1 in lung cancer chemoresistance, we amassed a collection of specimens from 18 SCLC patients that were treated with united platinum/etoposide chemotherapy. Surprisingly, the higher CILK1 expression in samples from treatment-resistant patients was revealed, compared with that of the sensitive/response group (Fig. 7A).

Subsequently, we generated two dual cisplatin (DDP)/epirubicin (EPI) resistant clones, H69R and H446R, which originated from SCLC cell lines, H69 and H446. We compared the expression of CILK1 in parental cells and their chemo-resistant derivatives, and revealed that total CILK1 and its phosphorylated form dramatically increased in chemo-resistant cells. Additionally, ERK1/2 were also activated in H69R and H446R cells, suggesting that CILK1/ERK1 signaling may play an important role in drug resistance (Fig. 7B). We also investigated whether short-term cisplatin or epirubicin treatment is sufficient to activate CILK1/ERK1 signaling, and the data confirmed that both CILK1 and ERK1 proteins were phosphorylated after epirubicin or cisplatin treatment for 3 to 12 hours (Fig. 7C). We silenced *CILK1* gene in H446R cells, and found that knockdown of CILK1 significantly inhibited the level of phosphorylated ERK1/2. In addition, CILK1-knockdown significantly reversed the drug resistance to cisplatin (Fig. 7D). Consistently, we observed the repression of ERK1/2 phosphorylation by CILK1-C30 in dose-dependent manner (Fig. 7E). Considering the united platinum/etoposide chemotherapy is the mainstay of treatment for SCLC patients 29, we tested the treatment effect of etoposide/cisplatin plus CILK1-C30 in H446 and H446R cells. The data indicated that CILK1-C30 greatly enhanced the killing effect of etoposide/cisplatin on tumor cells (Fig. 7F). CILK1-C30/platinum/etoposide robustly decreased the cell viability (Fig. 7G), and induced caspase-3 activation (Fig. 7H). Taken together, these results suggest that CILK1 plays a key role in promoting chemotherapeutic resistance in SCLC.

## Discussion

In this study, we identify the pro-oncogenic function of CILK1. As a MAPK- and CDK-like kinase, CILK1 is well established for its involvement in ciliogenesis and development of ciliopathies. CILK1 has been reported to participate in several important signaling such as Hedgehog and mTOR 30, 31, while the relationship between CILK1 and tumorigenicity remains poorly understood. Based on our large-scale analyses on breast cancer patient specimens, the expression of total and phosphorylated CILK1 protein was revealed to highly accumulate in tumor cells, and the expression of CILK1 protein has no specificity in breast cancer subtypes. Pharmacological inhibition or genetic silencing of CILK1 arrests tumor cell cycle progression and proliferation as well as reduces cancer cell viability. Over-expression of this gene enhances the growth potential and counteracts the cell apoptosis. Our data strongly support the conception that CILK1 plays as a driver of cancer progression. Furthermore, CILK1 was found to highly express in a series of other cancer types including lung and colon cancer, the anti-proliferation effect of CILK1 inhibition/silencing was also significant in small-cell lung cancer and colorectal cancer cell lines, reflecting its potential value for cancer treatment.

TNBC and SCLC both lack of specific molecular targets, cytotoxic chemotherapies remain the major treatment strategy in the clinical practice. Although part of these patients initially responds well to chemotherapies, once drug resistance occurs, the disease progression is invariably rapid. It has been implicated that CILK1 expression was elevated to confer cells with ability to counteract severe condition like nutrition deprivation 17. Therefore, we asked whether CILK1 is involved in chemotherapy resistance. Our data show that total and kinase-active CILK1 highly express in multiple chemotherapy-resistant cell lines and clinical therapy-resistant patients. Pharmacological inhibition or genetic silencing of CILK1 enhances chemotherapy regents-induced cytotoxicity in breast and lung cancer cells.

Our study identifies ERK1 as an important substrate and downstream signal of CILK1. The aberrant activation of Ras/Raf/MEK/ERK signaling is critical for cancer progression, which has been the subject of intensive pharmaceutical research and development 25. As key signaling nodes, ERK1/2 are attractive drug targets for cancer treatment. Although ERK1/2 phosphorylate plenty of downstream substrates that comprise over 160 proteins 32, quite limited upstream kinases of ERK1/2 have been identified so far. Actually, MEK1/2 have been reported to phosphorylate and activate ERK1/2 in most cases. Especially, MEK1/2 are the only two kinases that can phosphorylate Thr-202 of ERK1 and Thr-185 of ERK2. As we know, initially ERK1/2 are auto- and mono-phosphorylated at Tyr-204/187 in the activation loop, resulting in the basal kinase activity; but the full-active forms, which have approximately 500-fold activity than the basal forms, need the dual-phosphorylation at Thr-202/185 33. Combining multi-levels mass spectrum and biochemical analyses, we reveal CILK1 as an upstream kinase that directly phosphorylates ERK1 at Thr-202 residue. The isoforms ERK1 and ERK2 have significantly similar amino acid sequences, but exhibit distinctive physiological functions. Experimental evidences showed that knockout of ERK1 or ERK2 in the mice produced different genetic phenotypes 34. Although genetic silencing or pharmacological inhibition of CILK1 also inhibited Thr-185 phosphorylation of ERK2 based on the LC-MS/MS data, our kinase assay displayed that CILK1 was not able to catalyze the phosphorylation of ERK2, the decrease of ERK2 phosphorylation might be due to the indirect influence upon the loss of CILK1. The molecular mechanism why CILK1 specifically catalyze ERK1, but not ERK2, needs future clarification. We speculate it may be due to the structural difference between two proteins. Our data further indicate that CILK1 offers an alternative way for tumor cells to maintain the activation status of ERK1/2, as evidenced by the facts that only the treatment of MEK1/2 inhibitor in CILK1-silencing cells completely blocked the phosphorylation of ERK1/2, and over-expression of CILK1 was able to partially rescue the inhibition of phospho-ERK1/2 in the presence of MEK1/2 inhibitor. Additionally, inhibition of ERK1/2 repressed the enforcement of cell growth potential due to CILK1 over-expression, manifesting the functional dependence of CILK1 on ERK1. Our findings indicate that CILK1 cooperates with MEK to maintain the high-level of activation of ERK1 in tumor cells.

Our study explores potential strategies of pharmacological targeting towards CILK1. Since the dual phosphorylation of Tyr-159/Thr-157 stands for the activation of CILK1, we sought to screen the compounds that are able to block the modifications. As the high-level similarity of sequence between CILK1 and CDK1 proteins, we constructed the 3D structure around Tyr-159/Thr-157 residues in CILK1 protein by homology modelling and screened out tentative compounds based on molecular docking. CILK1-C30 and CILK1-C28, were found to efficiently block the dual phosphorylation, while were not able to affect the Thr-161 phosphorylation of CDK1. Importantly, their targeting specificity to CILK1 was confirmed by both CETSA and DARTS assays. And, both of them displayed potent cytotoxicity and anti-tumor efficiency, augmented the anti-cancer activity of chemotherapy reagents.

Overall, we reveal CILK1 as the prognostic marker and therapeutic target for cancer, and presented two CILK1-specific inhibitors supported by multiple evidences. Targeting CILK1/ERK1 signaling would provide a promising option for the treatment of cancer.

## Supplementary Material

Supplementary figures.

## Abbreviations

CILK1: Ciliogenesis-associated kinase 1
ERK1: Extracellular signal-regulated kinase 1
BC: Breast cancer
SCLC: Small-cell lung cancer
TNBC: Triple-negative breast cancer
CDK1: Cyclin dependent kinase 1
CETSA: Cellular thermal shift assays
DARTS: Drug affinity responsive target stability
NSG: NOD.Cg-Prkdc^scid^ Il2rg^tm1Wjl^/SzJ mice
