# Supplementary material for: Ciliogenesis-associated Kinase 1 Promotes Breast Cancer Cell Proliferation and Chemoresistance via Phosphorylating ERK1
Source: Int J Biol Sci. 2024 Apr 8;20(7):2403–21. doi: 10.7150/ijbs.87442 (PMC11077371; doi:10.7150/ijbs.87442)
Supplement: Supplementary file 1 — Supplementary figures. [file ijbsv20p2403s1.pdf]

Figure S1

A

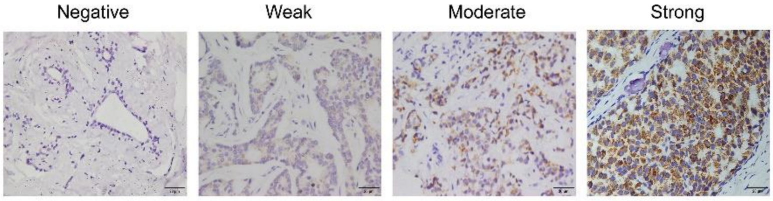

B

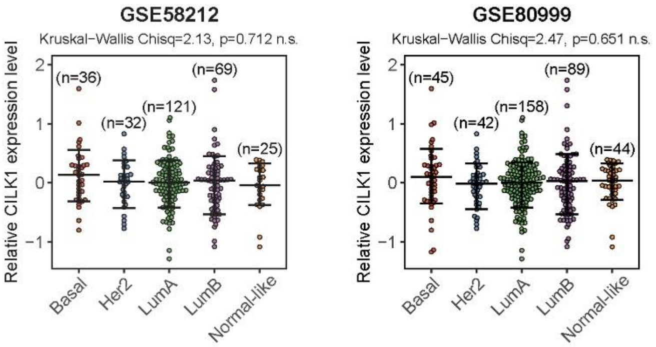

C

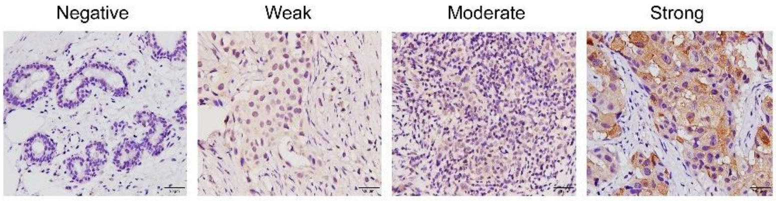

Supplemental Figure 1. CILK1 expression is up-regulated in breast cancer, related to Figure 1

(**A**) Example images showing the intensity of immunohistochemical staining of CILK1.

(**B**) The mRNA levels of CILK1 in GEO datasets that contained five subtypes of breast cancer, including BLBC, $HER2^+$, Lum-A, Lum-B and Normal-like.

(**C**) Images showing the intensity of immunohistochemical staining of phospho-CILK1 (Tyr-159).

Figure S2

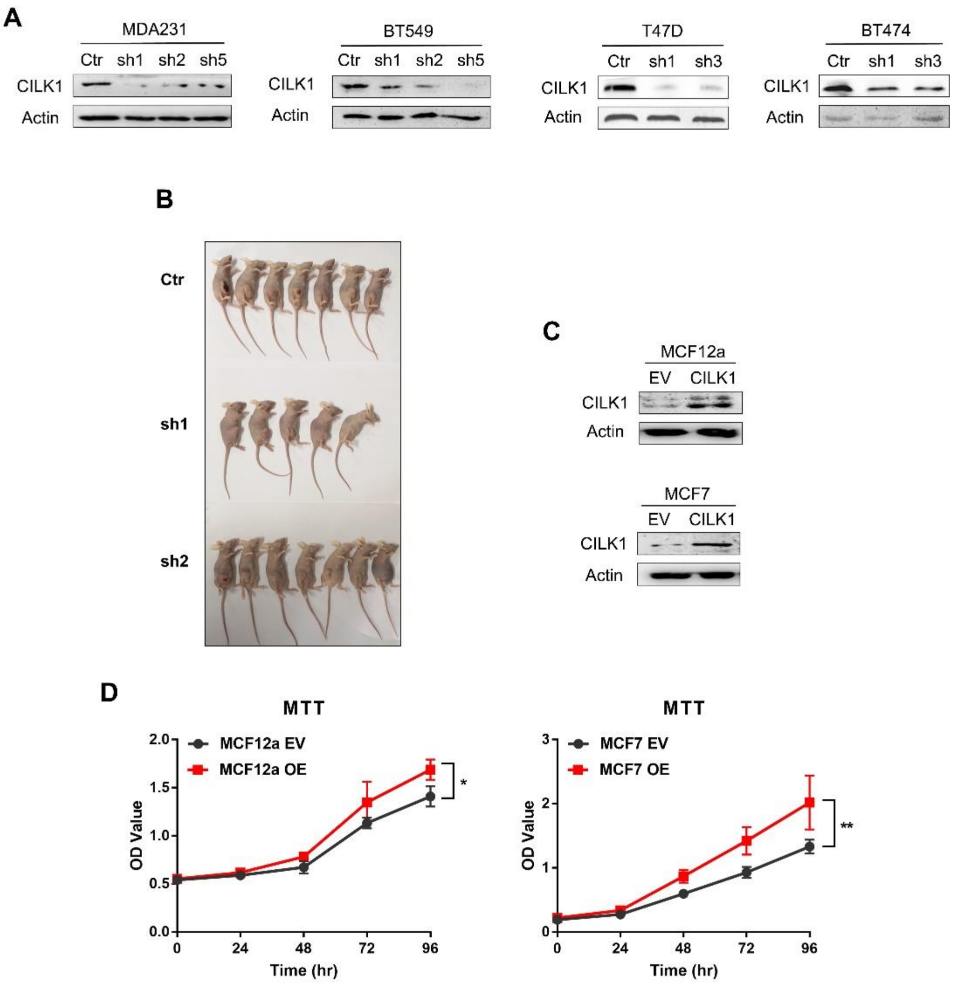

A
MDA231
Ctr sh1 sh2 sh5
CILK1
Actin
BT549
Ctr sh1 sh2 sh5
CILK1
Actin
T47D
Ctr sh1 sh3
CILK1
Actin
BT474
Ctr sh1 sh3
CILK1
Actin
B
Ctr
sh1
sh2
C
MCF12a
EV CILK1
CILK1
Actin
MCF7
EV CILK1
CILK1
Actin
D
MTT
MCF12a EV
MCF12a OE
OD Value
Time (hr)
0.0
0.5
1.0
1.5
2.0
0
24
48
72
96
*
MTT
MCF7 EV
MCF7 OE
OD Value
Time (hr)
0
1
2
3
0
24
48
72
96
**

Supplemental Figure 2. CILK1 is critical for breast cancer cell proliferation, related to Figure 2

(**A**) Western blot analysis for efficiency of CILK1-knockdown in breast cancer cell lines transfected with shRNA.

(**B**) Image of xenograft tumors in control and CILK1-knockdown groups.

(**C**) The over-expression status of CILK1 in MCF12a and MCF7 cells.

(**D**) MTT assay was performed to determine the effect of CILK1-overexpression on the proliferation of MCF12a (*left*) and MCF7 (*right*) cells. *$p < 0.05$, **$p < 0.01$.

Figure S3

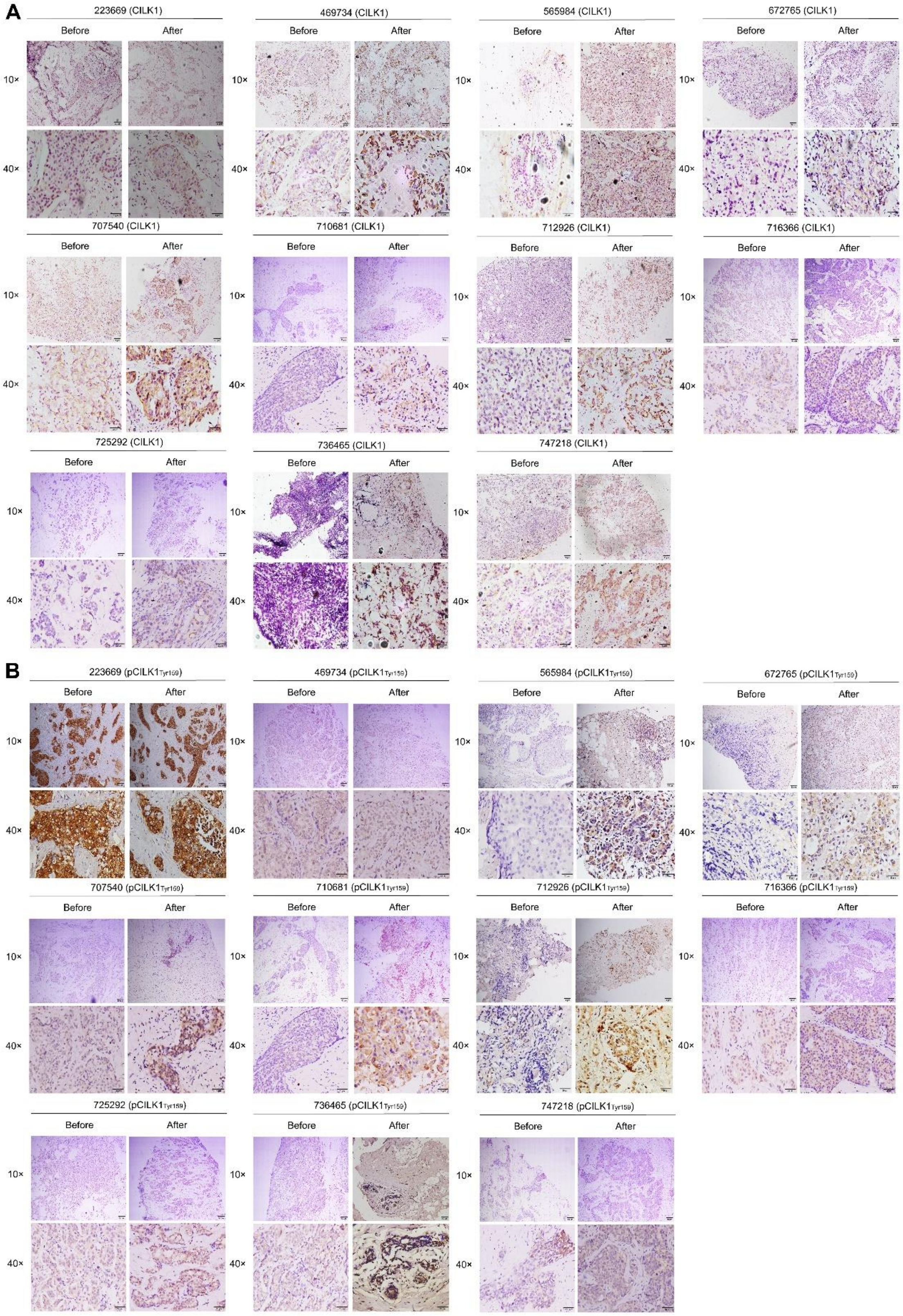
A
223669 (CILK1)
469734 (CILK1)
565984 (CILK1)
672765 (CILK1)
707540 (CILK1)
710681 (CILK1)
712926 (CILK1)
716366 (CILK1)
725292 (CILK1)
736465 (CILK1)
747218 (CILK1)
Before
After
10×
40×
B
223669 (pCILK1Tyr159)
469734 (pCILK1Tyr159)
565984 (pCILK1Tyr159)
672765 (pCILK1Tyr159)
707540 (pCILK1Tyr159)
710681 (pCILK1Tyr159)
712926 (pCILK1Tyr159)
716366 (pCILK1Tyr159)
725292 (pCILK1Tyr159)
736465 (pCILK1Tyr159)
747218 (pCILK1Tyr159)

Supplemental Figure 3. Elevated CILK1 expression confers resistance to chemotherapy, related to Figure 3

(**A**) IHC staining of CILK1 before and after paclitaxel treatment in breast cancer patients (n = 11).

(**B**) IHC staining of phospho-CILK1 (Tyr-159) before and after paclitaxel treatment in breast cancer patients (n = 11).

Figure S4

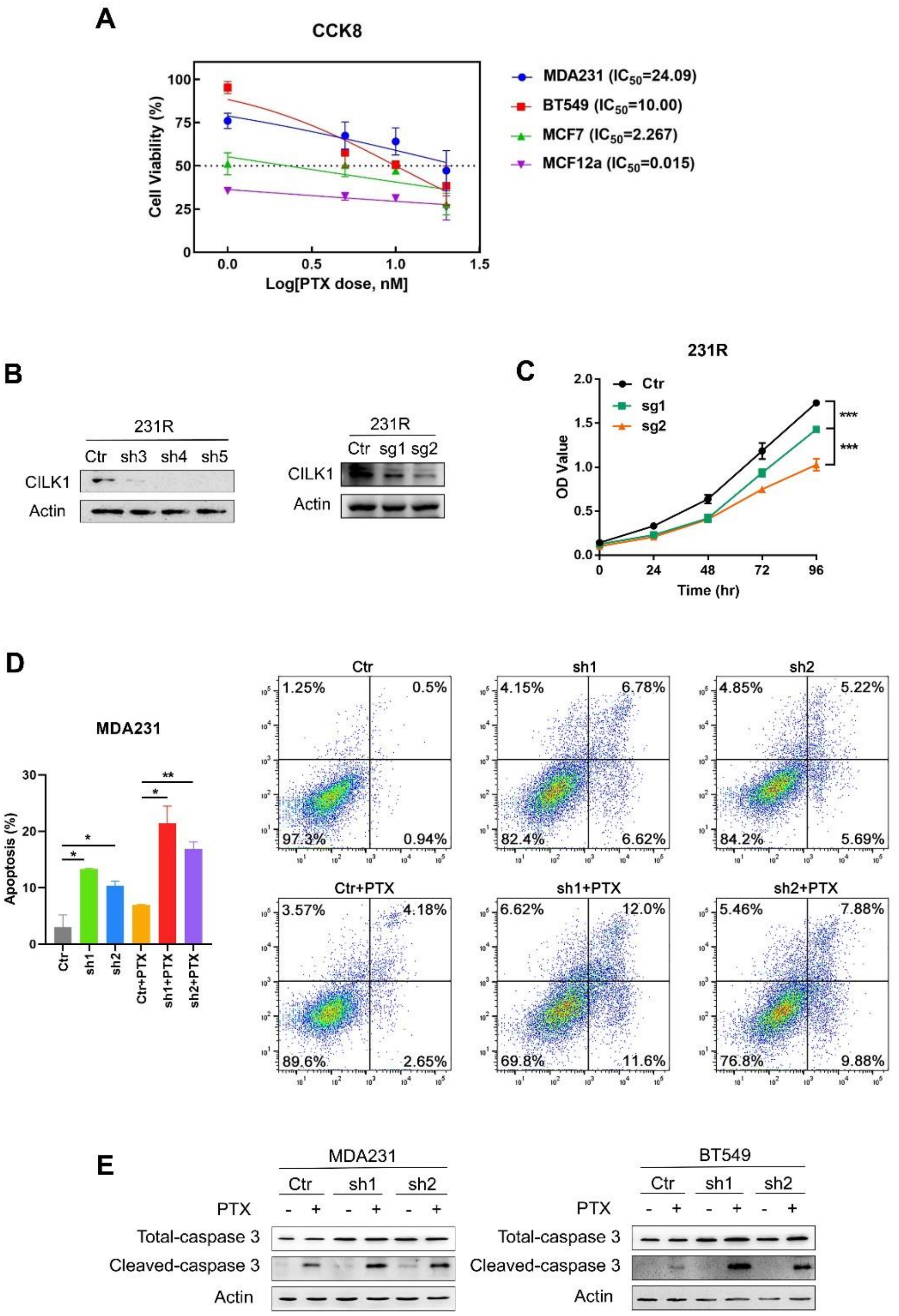
A
CCK8
Cell Viability (%)
Log[PTX dose, nM]
MDA231 (IC50=24.09)
BT549 (IC50=10.00)
MCF7 (IC50=2.267)
MCF12a (IC50=0.015)
B
231R
Ctr sh3 sh4 sh5
CILK1
Actin
231R
Ctr sg1 sg2
CILK1
Actin
C
231R
Ctr
sg1
sg2
OD Value
Time (hr)
***
***
D
MDA231
Apoptosis (%)
Ctr
sh1
sh2
Ctr+PTX
sh1+PTX
sh2+PTX
Ctr
1.25%
0.5%
97.3%
0.94%
sh1
4.15%
6.78%
82.4%
6.62%
sh2
4.85%
5.22%
84.2%
5.69%
Ctr+PTX
3.57%
4.18%
89.6%
2.65%
sh1+PTX
6.62%
12.0%
69.8%
11.6%
sh2+PTX
5.46%
7.88%
76.8%
9.88%
E
MDA231
Ctr sh1 sh2
PTX - + - + - +
Total-caspase 3
Cleaved-caspase 3
Actin
BT549
Ctr sh1 sh2
PTX - + - + - +
Total-caspase 3
Cleaved-caspase 3
Actin

Supplemental Figure 4. Elevated CILK1 expression confers resistance to chemotherapy, related to Figure 3

(A) IC_{50} values of 4 cell lines upon paclitaxel were measured by CCK8 assay.

(**B**) Western blot analysis for shRNA or CRISPR-Cas9 mediated knockdown efficiency of CILK1 in 231R cells.

(**C**) CCK8 assay was used to detect the effect of CILK1-knockdown by CRISPR-Cas9 on 231R cell proliferation.

(**D**) Annexin V-FITC flow cytometry assay was used to measure the pro-apoptotic effect of paclitaxel in control and CILK1-silencing MDA-MB-231 cells.

(**E**) Caspase-3 and cleaved-caspase-3 were detected in MDA-MB-231 and BT549 cells after treated with paclitaxel or DMSO for 48 hours.

Figure S5

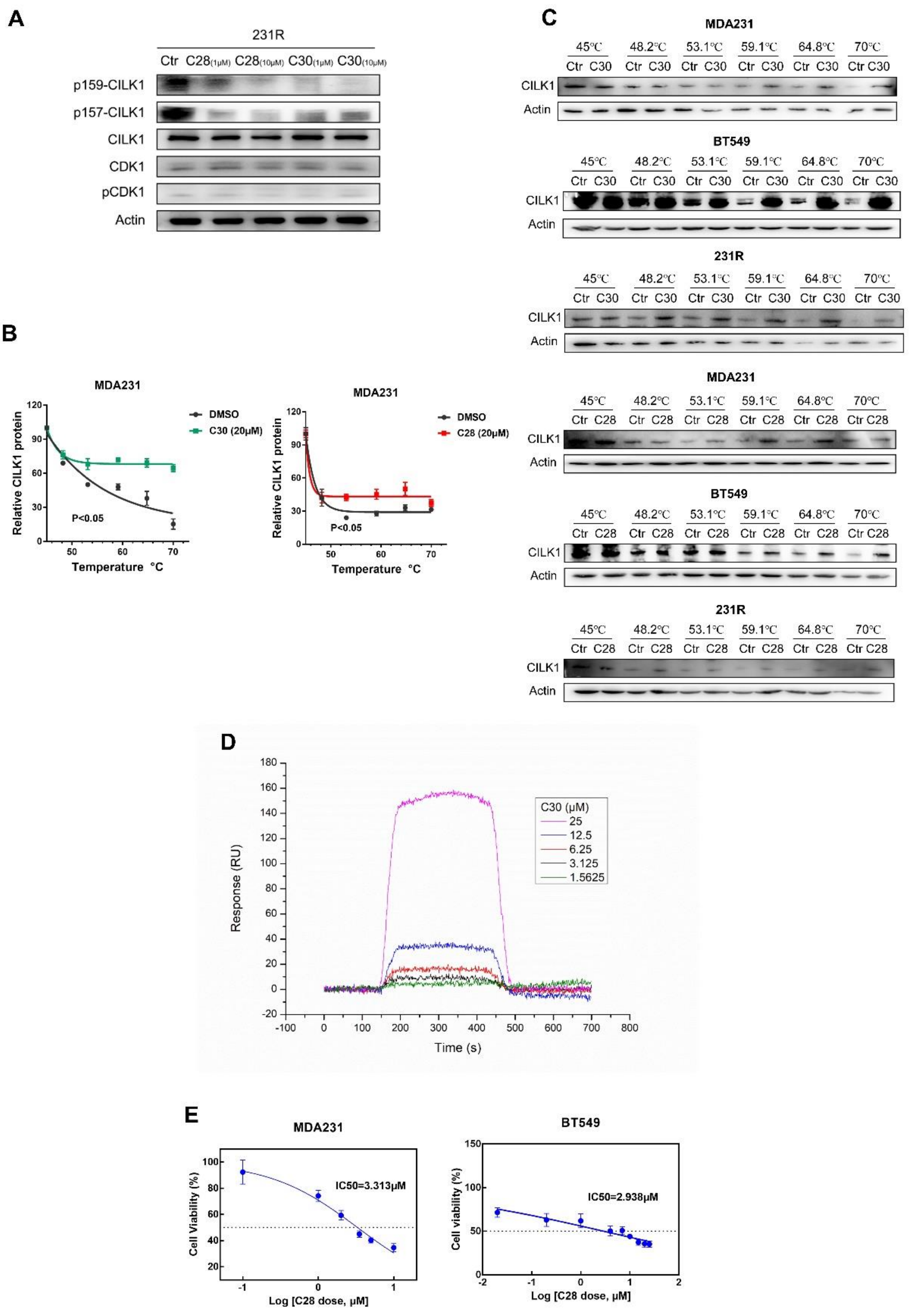
A
231R
Ctr C28(1μM) C28(10μM) C30(1μM) C30(10μM)
p159-CILK1
p157-CILK1
CILK1
CDK1
pCDK1
Actin
B
MDA231
DMSO
C30 (20μM)
Relative CILK1 protein
P<0.05
Temperature °C
MDA231
DMSO
C28 (20μM)
Relative CILK1 protein
P<0.05
Temperature °C
C
MDA231
BT549
231R
45℃ 48.2℃ 53.1℃ 59.1℃ 64.8℃ 70℃
Ctr C30
Ctr C28
CILK1
Actin
D
Response (RU)
Time (s)
C30 (μM)
25
12.5
6.25
3.125
1.5625
E
MDA231
Cell Viability (%)
IC50=3.313μM
Log [C28 dose, μM]
BT549
Cell viability (%)
IC50=2.938μM
Log [C28 dose, μM]

Supplemental Figure 5. Identification of selective inhibitors of CILK1, related to Figure 4

(**A**) Western blots were performed to detect the phosphorylation of CILK1 and CDK1 in 231R cells, which treated with DMSO or CILK1-C28 or CILK1- C30 for 24 h.

(**B**) Melt curves of CILK1 protein in CETSA analysis in MDA-MB-231 cells, treated with CILK1-C28 or CILK1-C30 or DMSO. The graph showed the quantification of CILK1 protein at different temperature points based on western blot.

(**C**) Representative western blots of CETSA were shown the effect of CILK1-C30/28 on thermal stabilization of CILK1 protein.

(**D**) SPR assay was used to examine the binding affinity between CILK1 and CILK1-C30.

(**E**) The inhibitory concentration curves of MDA-MB-231 and BT549 cells, treated with CILK1-C28. And the IC_{50} values were calculated using GraphPad Prism.

Figure S6

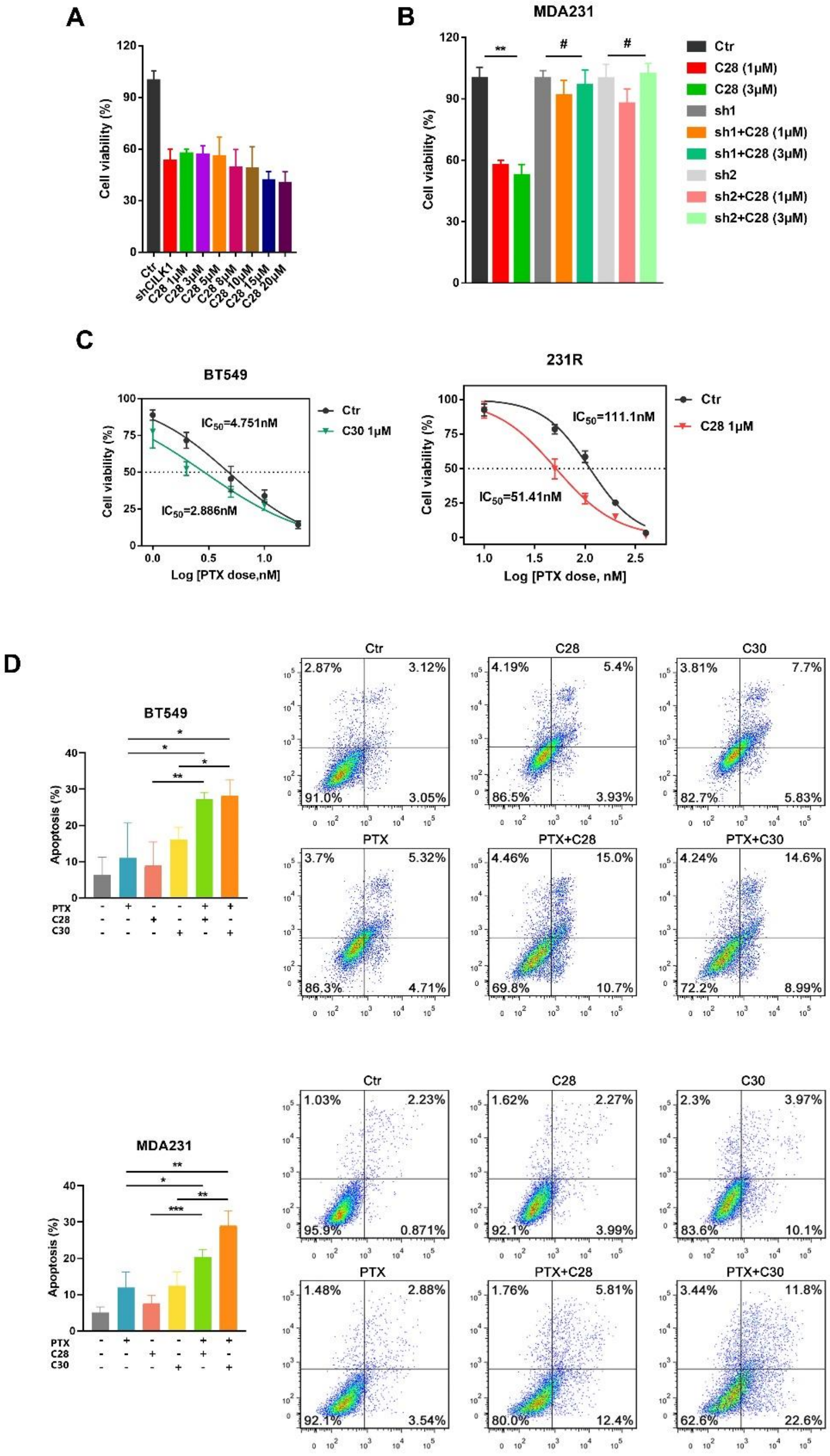
A
Cell viability (%)
Ctr
shCILK1
C28 1μM
C28 3μM
C28 5μM
C28 8μM
C28 10μM
C28 15μM
C28 20μM
B
MDA231
Cell viability (%)
Ctr
C28 (1μM)
C28 (3μM)
sh1
sh1+C28 (1μM)
sh1+C28 (3μM)
sh2
sh2+C28 (1μM)
sh2+C28 (3μM)
C
BT549
IC50=4.751nM
IC50=2.886nM
Ctr
C30 1μM
Log [PTX dose,nM]
231R
IC50=111.1nM
IC50=51.41nM
Ctr
C28 1μM
Log [PTX dose, nM]
D
BT549
Apoptosis (%)
PTX
C28
C30
Ctr
C28
C30
PTX
PTX+C28
PTX+C30
MDA231
Apoptosis (%)

Supplemental Figure 6. Identification of selective inhibitors of CILK1, related to Figure 4

(**A**) CCK8 assay was done in control or CILK1-knockdown MDA-MB-231 cells, and various concentrations of C28 were used to treat control MDA-MB-231 cells for 48 h.

(**B**) CCK8 assay was done in control or CILK1-knockdown MDA-MB-231 cells, treated with vehicle or C28 (1 μM or 3 μM) for 48 h. Data were presented as mean ± SD; **p < 0.01, #p > 0.05; one-way ANOVA.

(**C**) IC_{50} values of breast cancer cells for paclitaxel were measured by CCK8 assay. Various concentrations of paclitaxel were used to treat cells, pre-treatment with CILK1-C30 or C28.

(**D**) Flow cytometry was performed to measure the pro-apoptotic effect of CILK1-C30/28 in MDA-MB-231 and BT549 cells, combined with paclitaxel. Data were presented as mean ± SD, three independent experiments were performed (**p < 0.01, ***p < 0.001, one-way ANOVA).

Figure S7

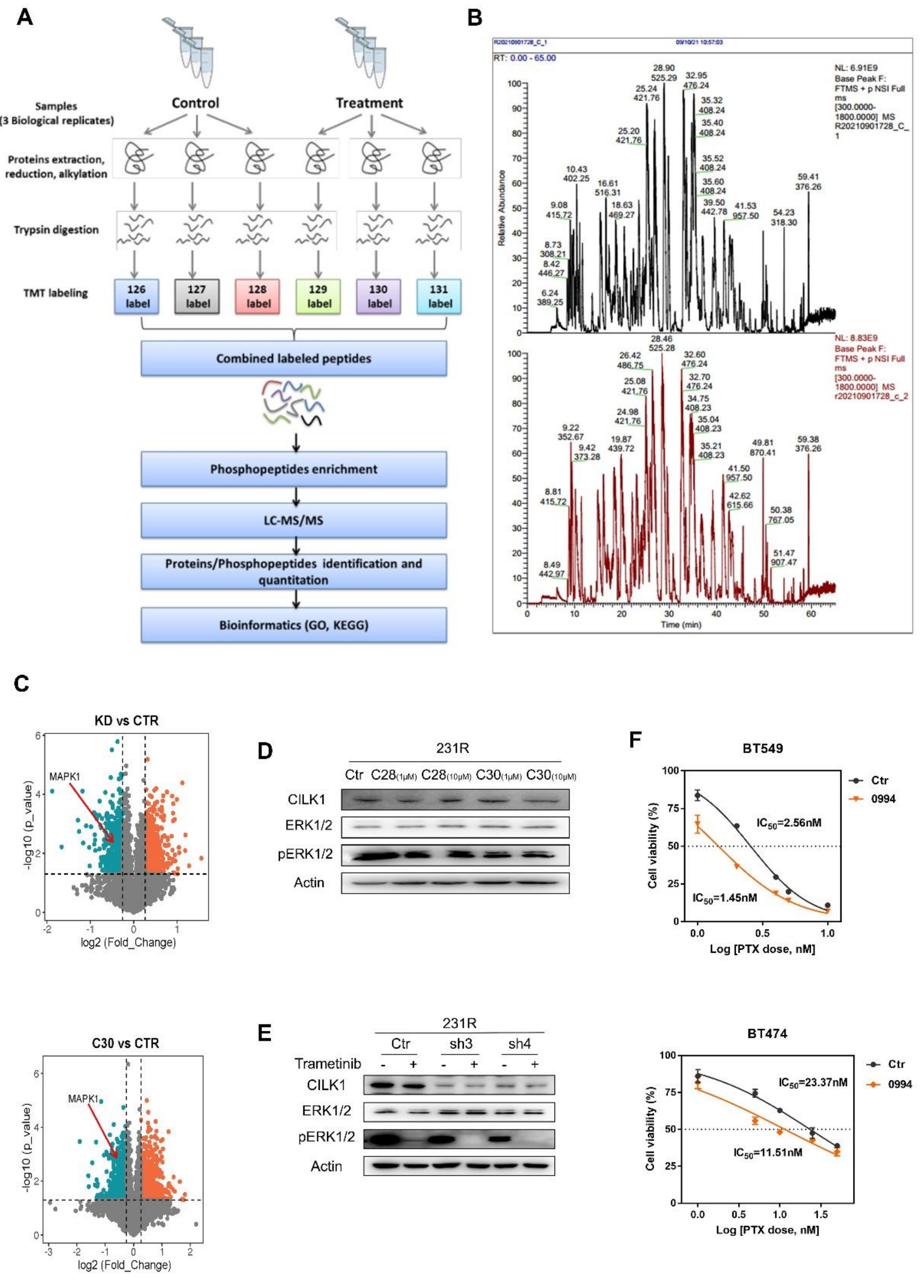
A
Control
Treatment
Samples (3 Biological replicates)
Proteins extraction, reduction, alkylation
Trypsin digestion
TMT labeling
126 label
127 label
128 label
129 label
130 label
131 label
Combined labeled peptides
Phosphopeptides enrichment
LC-MS/MS
Proteins/Phosphopeptides identification and quantitation
Bioinformatics (GO, KEGG)
B
RT: 0.00 - 65.00
Relative Abundance
Time (min)
C
KD vs CTR
MAPK1
-log10 (p_value)
log2 (Fold_Change)
C30 vs CTR
D
231R
Ctr
CILK1
ERK1/2
pERK1/2
Actin
E
Trametinib
sh3
sh4
F
BT549
Ctr
0994
IC50=2.56nM
IC50=1.45nM
Cell viability (%)
Log [PTX dose, nM]
BT474
IC50=23.37nM
IC50=11.51nM

Supplemental Figure 7. CILK1 directly phosphorylates ERK1, related to Figure 5

(**A**) Scheme of LC-MS/MS experiment to identify CILK1 substrates.

(**B**) LC-MS/MS base peak chromatograms of all proteins.

(**C**) Volcano plots of the quantitative TMT-based proteomic analysis, which indicated MAPK1 as one of the differential phospho-proteins. The data of CILK1-knockdown or CILK1-C30-treatment were shown respectively. Blue and red dots represented significantly down- and up-regulated phospho-proteins ($p < 0.05$).

(**D**) Western blot was performed to detect the expression of ERK1/2 and phospho-ERK1/2 in control and CILK1-C30/28 treated 231R cells.

(**E**) Western blot was used to detect the levels of CILK1, ERK1/2 and phospho-ERK1/2 in control and CILK1-knockdown 231R cells, which treated with vehicle or trametinib (10 nM) for 30 min.

(**F**) IC_{50} values of BT549 and BT474 cells for paclitaxel were measured by CCK8 assay, cells were treated with GDC-0994 (150 nM) for 48 h.

Figure S8

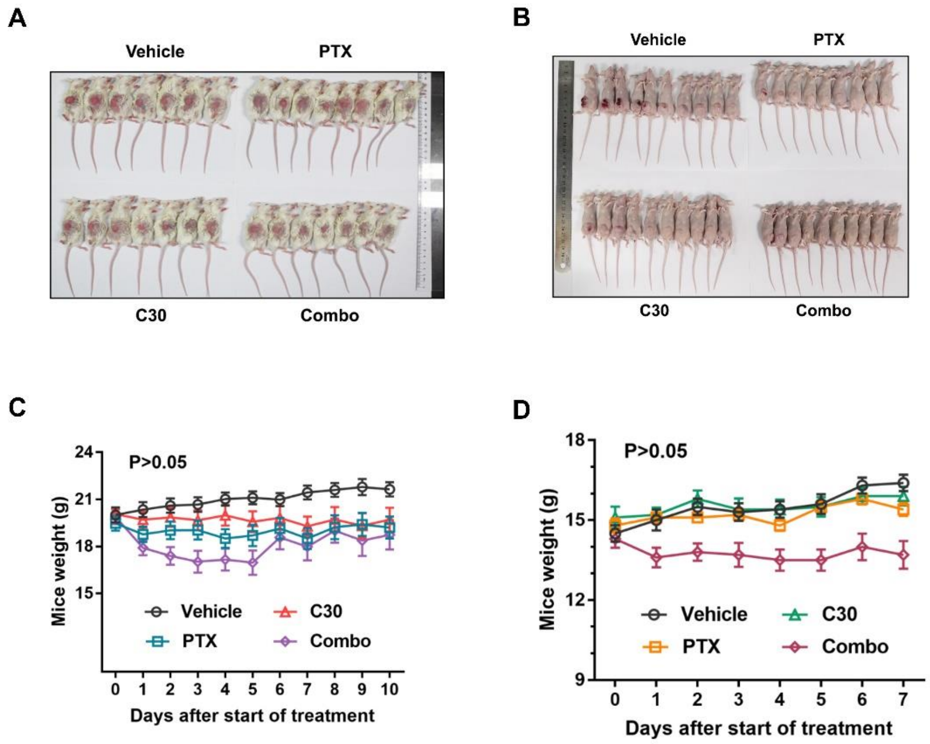

A
Vehicle
PTX
C30
Combo
B
Vehicle
PTX
C30
Combo
C
P>0.05
Mice weight (g)
Days after start of treatment
Vehicle
C30
PTX
Combo
D
P>0.05
Mice weight (g)
Days after start of treatment
Vehicle
C30
PTX
Combo

Supplemental Figure 8. Pharmacological inhibition of CILK1 in TNBC mice models, related to Figure 6

(A-B) Images of the mice tumor for PDX (A) and CDX (B) models.

(C-D) Body weight of the mice during the treatment for PDX (C) and CDX (D) models.
